# Genetic Variation in Human Gene Regulatory Factors Uncovers Regulatory Roles in Local Adaptation and Disease

**DOI:** 10.1093/gbe/evz131

**Published:** 2019-06-22

**Authors:** Álvaro Perdomo-Sabogal, Katja Nowick

**Affiliations:** Human Biology Group, Department of Biology, Chemistry and Pharmacy, Institute for Zoology, Freie Universität Berlin, Germany

**Keywords:** transcription factor, positive selection, *Krüppel*-associated box (KRAB-ZNF) cluster, schizophrenia

## Abstract

Differences in gene regulation have been suggested to play essential roles in the evolution of phenotypic changes. Although DNA changes in *cis-*regulatory elements affect only the regulation of its corresponding gene, variations in gene regulatory factors (*trans*) can have a broader effect, because the expression of many target genes might be affected. Aiming to better understand how natural selection may have shaped the diversity of gene regulatory factors in human, we assembled a catalog of all proteins involved in controlling gene expression. We found that at least five DNA-binding transcription factor classes are enriched among genes located in candidate regions for selection, suggesting that they might be relevant for understanding regulatory mechanisms involved in human local adaptation. The class of KRAB-ZNFs, zinc-finger (ZNF) genes with a *Krüppel*-associated box, stands out by first, having the most genes located on candidate regions for positive selection. Second, displaying most nonsynonymous single nucleotide polymorphisms (SNPs) with high genetic differentiation between populations within these regions. Third, having 27 KRAB-ZNF gene clusters with high extended haplotype homozygosity. Our further characterization of nonsynonymous SNPs in ZNF genes located within candidate regions for selection, suggests regulatory modifications that might influence the expression of target genes at population level. Our detailed investigation of three candidate regions revealed possible explanations for how SNPs may influence the prevalence of schizophrenia, eye development, and fertility in humans, among other phenotypes. The genetic variation we characterized here may be responsible for subtle to rough regulatory changes that could be important for understanding human adaptation.

## Introduction

The molecular basis of phenotypic divergence between species and populations is still far from being fully understood. In particular, within short evolutionary time scales, changes in gene expression play crucial roles in the diversification of phenotypical traits (Bornberg-Bauer et al. 2010; Nowick et al. 2011; Albert and Kruglyak 2015). Alterations in gene expression are mainly caused by sequence variations in the promoter regions of genes (*cis*-changes) or in the factors that regulate the expression of other genes (*trans*-changes). Gene regulatory factors (GRFs) include proteins that bind directly to DNA (Wingender et al. 2015), cofactors that bind to transcription factors (TFs) bound to DNA, and histone modifying enzymes (Li et al. 2015). Another type of regulatory molecules, noncoding RNAs, also plays a role in gene regulation (Zhu et al. 2013). Variations in the expression levels, timing, and tissue-specificity coupled with sequence and structural changes in GRFs are important components of the genotype–phenotype map (Emerson and Li 2010).

Several studies have already investigated *cis*-changes among primates and anatomically modern human (AMH), suggesting that a substantial fraction of the phenotypic differences we observe across species is probably explained by specific variation in gene regulatory mechanisms (Wray 2007; Wittkopp and Kalay 2012; Lappalainen et al. 2013; Perdomo-Sabogal et al. 2016). GRF changes have also been documented in primates, demonstrating that some classes of GRFs have rapidly expanded and evolved in the human lineage (Nowick et al. 2011; Lukic et al. 2014; Fyon et al. 2015; Zhang et al. 2015). For instance, zinc-finger (ZNF) genes with a *Krüppel*-associated box (KRAB-ZNF), a subclass of C2H2 ZNF genes, have undergone different evolutionary processes than other non-KRAB C2H2 genes. These processes include in situ tandem duplications that resulted in an extensive expansion of KRAB-ZNF genes in mammals and a broad repertoire of lineage-specific genes, paralog diversification through positive selection and changes in the DNA-binding specificity of the ZNF arrays, and an excess of rapidly evolving genes in primate species (Hamilton et al. 2003; Huntley et al. 2006; Emerson and Thomas 2009; Lorenz et al. 2010). Several KRAB-ZNF genes are considered to be human-specific (Nowick et al. 2010). Other KRAB-ZNFs exhibit functional differences in their DNA-binding domains between humans and chimpanzees (Nowick et al. 2011) Further, ZNF genes associated with adaptive mechanisms to downregulate inherited endogenous retroelements in humans have been described (Thomas and Schneider 2011; Jacobs et al. 2014; Lukic et al. 2014). The rapid evolution of several GRFs in the primate lineage (Nowick et al. 2011; Lukic et al. 2014; Fyon et al. 2015; Zhang et al. 2015) suggests that GRFs may also exhibit elevated differentiation across human populations and potentially contribute to local adaptation.

Genes that control the adaptation of phenotypes to environmental fluctuations are subject to positive selection. Several candidate genes that were likely target of natural selection in humans have already been identified (Sabeti et al. 2007; Nielsen et al. 2009; Pickrell et al. 2009; Grossman et al. 2013). However, a detailed exploration of how natural selection might have influenced the diversity of GRF genes and contributed to human phenotypic variation and local adaptation has not been attempted yet. In this study, we analyzed signatures of positive selection in several classes of GRFs in three human populations. We also tested whether these functional classes exhibit significant differences with respect to their past exposure to positive selection. To this end, we built a comprehensive catalog of human genes coding for proteins involved in transcriptional regulation by including and manually curating information from existing databases and inventories. Our results show that genes coding for GRFs are overrepresented in genomic regions associated with a signal of positive selection. We also found that many KRAB-ZNF gene clusters exhibit reduced genetic variation and extended haplotype homozygosity (EHH) at population-specific level. Leveraging available functional information and genetic variation from European and Asian populations allowed us to generate testable hypotheses on reasons for the higher prevalence of schizophrenia outside Europe. In addition, a similar analysis between European, Asian, African, Iberian, and Latin American populations, as well as archaic humans, support the potential roles of a human-specific KRAB-ZNF gene in developmental changes of the eye and adaptation to high/low ultraviolet (UV) exposure. Our examples also provide insights into how genetic variation might subtly tweak GRFs’ binding affinity and their regulatory activity, thus allowing for population-specific phenotypic variation.

## Materials and Methods

### Building the GRF Gene Catalog

We put together GRF genes from seven different lists previously published for humans. These gene sets included TF transcriptome and sequence evolution studies, TF inventories (Messina et al. 2004; Vaquerizas et al. 2009; Ravasi et al. 2010; Nowick et al. 2011; Corsinotti et al. 2013; Tripathi et al. 2013), and functional classifications (Wingender et al. 2015) (supplementary table S1, Supplementary Material online). Producing the GRF catalog reported here, which contains 3,344 out of 28,026 genes from the human genome (Hg19), involved multiple strategies (see supplementary material, Supplementary Material online).

### Identifying Candidate GRFs for Selection in Three AMHs Populations

Using whole genome sequencing data from 1000G project (1000 Genomes Project Consortium 2012) and the data from 1000 Genomes Selection Browser 1.0 (Pybus et al. 2014), we identified candidate genomic regions that exhibit genetic variation agreeable with signatures of positive selection. We mainly explored three AMHs populations: Utah Residents with Northern and Western European Ancestry (CEU), Han Chinese in Bejing (CHB), and Yoruba in Ibadan (YRI). We considered that the samples from these three human populations sufficiently represent broader geographical regions of ancestry, as suggested by Lu and Xu (2013). To better understand how natural selection may have shaped the diversity we currently observe for GRF genes in these three different human populations, we first analyzed the results obtained from three different methods (CLR, XP-CLR, and XP-EHH) for detecting positive selection in three human populations (Pybus et al. 2014). As additional strategy to complement our findings, we also included the *F*_ST_ statistics; a test for measuring population genetic differentiation. The data cover 83% of the GRF genes we cataloged, whereas for the remnant 17% there was not information available.

By using ranked score values based in the genome-wide distribution obtained for each population (Pybus et al. 2014), and considering the complexity of human demography, we defined as candidates for positive selection all GRFs located in the 5% most extreme regions in all three tests designed for detecting positive selection (intersection between CLR, XP-CLR, and XP-EHH). Defined as outlier approach, reporting extreme values has been used in previous scans for detecting selection in humans (Voight et al. 2006; Pybus et al. 2014; Huber et al. 2016), despite no additional tests have been implemented in Voight et al. (2006), Huber et al. (2016), or here to identify if the most extreme values we report can indeed be considered as outliers with respect to a parametric distribution. Our rationale behind this is that scores found in the upper tail of the distribution are expected to indicate deviations from neutrality and suggests regions/genes that may have undergone positive selection.

### GRF Overrepresentation

We evaluated if GRFs are enriched among the top 5% of all human protein-coding genes for each one of the three statistical tests for detecting positive selection (CLR, XP-CLR, and XP-EHH), and genetic differentiation (*F*_ST_) in any of three human populations (CEU, CHB, and YRI). To do this, we used our catalog of GRFs and generated two sets of genes, GRF and non-GRF genes. We then performed a Fisher’s exact test to evaluate if GRF genes presented more extreme rank scores than other (non-GRFs) human genes. We then corrected the empirical *P* value using Bonferroni correction for multiple testing.

### Regions with Biased Patterns of Variation and Long EHH within KRAB-ZNF Gene Clusters

Using the annotation for KRAB-ZNF gene clusters described in Huntley et al. (2006), we first searched for regions with high CLR, XP-CLR, and XP-EHH (ranked scores >1.3, empirical *P* of 0.05). For regions that exhibited long EHH results, we then tested whether the extension of uninterrupted single nucleotide polymorphisms (SNPs) with high XP-EHH rank scores was more than what could be expected by chance. We measured where the linkage disequilibrium decay falls below *r*^2^ = 0.1 in the empirical data (YRI ∼ 20 kb, CEU, and CHB ∼ 30 kb). We then performed 1,000 random samplings, of genomic regions of the same size as the KRAB-ZNF regions that display EHH larger (>50 kb) and counted the number of uninterrupted SNPs with significant XP-EHH rank scores. We then implemented Bonferroni correction for multiple testing. By using a similar strategy, we additionally tested if the recombination rates observed for those 32 KRAB-ZNF clusters we detected as initial candidates were smaller than expected by chance. We randomly sampled genomic regions of the same size 1,000 times, calculated the mean of the recombination rates, and measured how often these were smaller than the mean observed in those KRAB-ZNF regions. We corrected for multiple testing (Bonferroni method). Afterward, we defined candidate regions for positive selection between the start and end positions of genomic regions with one or more haplotypes at high frequency, reduced variability and EHH larger than 50 kb.

To identify if the patterns of variation we observed in the 32 candidate KRAB-ZNF clusters resemble scenarios of selective sweeps and to evaluate whether the observed scores calculated from the empirical data are unusually high when compared with expectations under neutrality, we simulated data using Cosi2 simulator (Shlyakhter et al. 2014). Cosi2 simulator already considers a demographic model that is consistent for demography for these three human populations (Schaffner et al. 2005). We additionally adjusted this best fitted model following the adjustments implemented by Pybus et al. (2015), which tuned the model to generate sequence data that resemble the linkage disequilibrium decay, genetic variation and site frequency spectrum. We then performed 1,000 simulations for each of the 32 KRAB-ZNF clusters that exhibited multiple haplotypes at high frequency. We also performed singleton thinning following the values obtained by Pybus et al. (2015) to reduce the bias against rare variants due to the low coverage of the data (48% of the global singletons were removed). We finally compared the empirical data from these 32 candidate regions versus the simulated variation generated under neutrality.

## Results

### A Comprehensive Catalog of Human GRFs for Studying Regulatory Evolution

To investigate the roles of GRFs during human evolution, we assembled the most up-to-date catalog of GRF genes combining the information from eight studies (table 1 and supplementary Methods.pdf, Supplementary Material online). In total, our catalog encompasses 3,344 genes (supplementary table S1, Supplementary Material online).

**Table 1:** Composition of 3,344 GRF Genes Considered in This Study (see Supplementary Material, Supplementary Material Online, for Selection Criteria) and the Sources Where These Genes Were Previously Cataloged

Extant Inventories Human GRFs	Genes Included	% Included
Messina et al. (2004)	1,640	84.1
Vaquerizas et al. (2009)	1,804	96.6
Ravasi et al. (2010)	1,734	87.2
Nowick et al. (2011)	572	96.5
Corsinotti et al. (2013)	339	96.3
Tripathi et al. (2013)	2,998	92.3
Karolchik et al. (2012)	2,225	86.6
Wingender et al. (2015)	1,506	99.8
Present work	3,344	100

Following the detailed and curated classification of DNA-binding TF genes (Wingender et al. 2015), we functionally grouped 1,509 GRF genes into 40 TF classes. ZNF genes are by far the most abundant class (807 genes) that further breaks down into ten subclasses, of which the KRAB-ZNF (410 genes) and non-KRAB C2H2 (280 genes) are the most abundant. They are followed by the classes of Homebox Domain (229 genes) and basic Helix-Loop-Helix (bHLH, 107 genes) (supplementary fig. S1, Supplementary Material online).

### GRF Genes Are Overrepresented in Candidate Regions for Positive Selection

To identify GRFs located in genomic regions potentially subjected to positive selection, we analyzed the genome-wide rank scores for four different methods: the multiple-locus composite likelihood ratio (CLR) (Nielsen et al. 2009), cross-population CLR (XP-CLR) (Chen et al. 2010), cross-population extended haplotype homozygosity (XP-EHH) (Sabeti et al. 2007), and *F*_ST_ (Weir and Cockerham 1984), in three human populations (CEU, CHB, and YRI). Regions obtaining the highest scores with these methods display patterns of variation consistent with genetic differentiation across populations and putative positive selection. GRFs are enriched among the top 5% of the ranked scores with the window based methods CLR and XP-CLR for most populations and pairwise comparisons (Fisher’s exact test, Bonferroni corrected *P* < 0.01) (table 2). With the XP-EHH test we found either depletion or no difference in ranked score distribution between GRFs and other genes when comparing CEU and CHB versus YRI. It is possible that the lower number of haplotype blocks that is characteristic of sub-Saharan populations (Gabriel et al. 2002; International HapMap Consortium et al. 2007) may have caused this observed depletion. Importantly, there was no significant difference between the distributions of the recombination rates between GRF and non-GRF genes (Kolmogorov–Smirnov test; *D* = 0.019; *P* = 0.18) and only a very small, albeit significant, correlation between gene length and the rank score (Spearman rank correlation, *P* < 2.2e-16, *ρ* = 0.009) at population level (supplementary Methods.pdf, Supplementary Material online). This indicates that differences in recombination rates and gene length probably did not bias our findings.

**Table 2:** Association between GRF and non-GRF genes and the level of significance for three statistics for identifying candidate regions for positive selection and Measuring genetic differentiation (F_*ST*_).

Test	Populations	Fisher Exact Test (Bonferroni Corrected *P*)	Odds Ratio	Feature
CLR	CEU	3.96E-15	1.207	Enrichment
	CHB	9.72E-02	1.066	No difference
	YRI	2.70E-07	1.132	Enrichment
XP-CLR	CEU versus CHB	3.96E-04	1.145	Enrichment
	CEU versus YRI	1.58E-14	1.278	Enrichment
	CHB versus CEU	3.42E-10	1.235	Enrichment
	CHB versus YRI	8.64E-08	1.203	Enrichment
	YRI versus CEU	4.50E-09	1.219	Enrichment
	YRI versus CHB	1	1.01	No difference
XP-EHH	CEU versus CHB	3.96E-15	1.367	Enrichment
	CEU versus YRI	3.96E-15	0.906	Depletion
	CHB versus CEU	1.73E-03	1.043	No difference
	CHB versus YRI	3.96E-15	0.896	Depletion
	YRI versus CEU	1	1.016	No difference
	YRI versus CHB	1	0.988	No difference
*F*_ST_	CEU versus CHB	1.04E-01	0.971	No difference
	YRI versus CEU	1.19E-01	1.023	No difference
	YRI versus CHB	1	1.013	No difference

We next selected as candidates for positive selection, per each population, GRF genes which are found among the top 5% of the genome-wide rank score distribution in all three selection-detection methods (CLR, XP-CLR, and XP-EHH). This yielded 902 GRF genes for CEU, 759 GRF genes for CHB, and 1,697 GRF genes for YRI (supplementary table S2, Supplementary Material online). Subsequently, we intersected the lists across populations and found that 306 GRF genes are found as candidates in all three populations (supplementary fig. S2, Supplementary Material online). Although YRI has most population-specific candidates (892 GRFs, 53%), CEU and CHB display a bigger overlap among pairs of comparisons (between 74% and 76% of the total candidates, respectively).

**Fig. 1.**
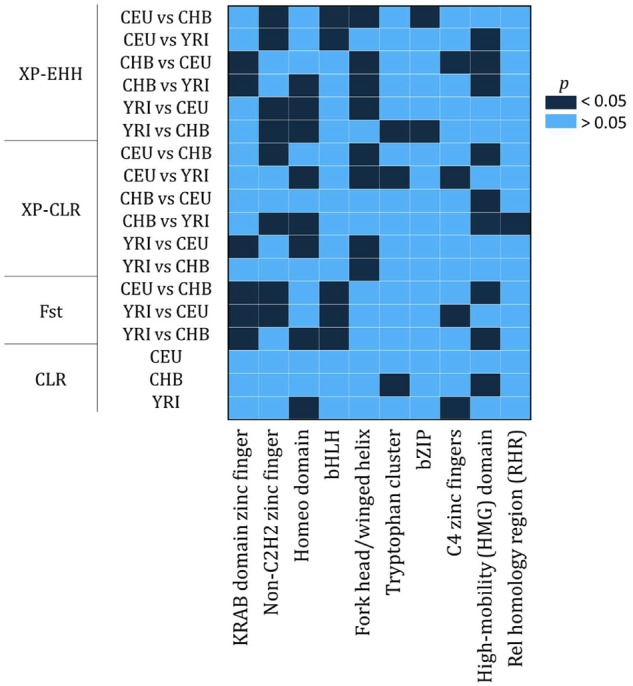
—Enrichment analyses for genes from the ten largest DNA–binding GRFs classes located in regions exhibiting high scores for four methods for detecting candidate regions for positive selection and one for measuring genetic differentiation. This heatmap shows the results from the Fisher’s exact test after correcting for multiple testing by using the Bonferroni correction for each population or cross-population comparison, respectively.

We next evaluated, whether any of the 40 GRF classes was enriched among the top 5% of candidates with highest rank scores. Although the enriched classes (Fisher’s exact test, Bonferroni corrected *P* < 0.05) differed slightly depending on the method and population (fig. 1 and supplementary table S3, Supplementary Material online), five of the ten largest GRF classes were repeatedly found to be overrepresented: KRAB-ZNFs, non-C2H2, Homeo domain, High-mobility HMG, and Forkhead box TFs (fig. 1).

In total, these five GRF classes contain 180 candidates for positive selection. One hundred and twenty-one of these genes belong to the TF class C2H2 (68 non-KRAB C2H2 and 53 KRAB-ZNF candidates). They are followed by Homeo domain with 31, Fork head/winged helix with 19, and High-mobility-HMG domain with nine candidates (supplementary table S4, Supplementary Material online). Even though larger classes had more genes located within the top 5% of the highest scores, percentagewise they are also overrepresented: non-KRAB C2H2 (24%), KRAB-ZNF (13%), Homeo domain (13%), Forkhead box (28%), and High-mobility HMG (21%). Out of these 180 GRF, only 21 genes (12%) have also been listed in previous scans for selection (Sabeti et al. 2007; Pickrell et al. 2009; Metspalu et al. 2011; Grossman et al, 2013; Huber et al. 2016), six of them being non-KRAB C2H2 (*ZFAT*, *ZBTB41*, *ZNF827*, *IKZF2*, *ZNF438*, and *ZBTB20*) and four KRAB-ZNF (*ZNF546*, *ZNF780A*, *ZNF780B*, and *ZNF492*) genes (supplementary table S5, Supplementary Material online). As a group, these five GRF classes are of relevance for processes like embryogenesis, development, chromatin modification, DNA replication and repair, among others (table 3).

**Table 3:** Main Biological Roles of the Five Repeatedly Enriched GRF Classes within the Top 5% of Putative Regions for Positively Selection

GRF Family	Examples of Main Regulatory Roles
Forkhead boxes	Cell growth, proliferation, differentiation, and longevity; embryonic development; cell migration; organ development, T-lymphocyte proliferation (Jonsson and Peng 2005; Tuteja and Kaestner 2007a, 2007b).
C2H2	Establishment of the chromosomal architecture; embryonic development, cell differentiation and proliferation, regulation of the cell cycle and apoptosis (Fedotova et al. 2017).
KRAB-ZNF	Recruitment of TRIM28/KAP-1 for repression of gene expression, epigenetic silencing; early embryonic development; repression of ERVs and transposable elements; establishment of postzygotic reproductive isolation (speciation) (Nowick et al. 2013; Wolf et al. 2015; Kapopoulou et al. 2016; Fedotova et al. 2017). Function of most of them is unknown yet.
Homeo domain	Body plan specification during embryogenesis, regulation of axial patterning, segment or cell identity and proliferation; formation and cell fate determination in metazoan development, crucial for normal temporospatial limb and organ development (Banerjee-Basu and Baxevanis 2001).
High-mobility HMG	Bind temporally to nucleosomes to modify local chromatin architecture; DNA replication and repair; architectural proteins of nucleus and mitochondrial DNA; signaling regulators in the cytoplasm and as inflammatory cytokines (Wang et al. 1999; Lim et al. 2004; Malarkey and Churchill 2012).

### High Nonsynonymous Genetic Differentiation within KRAB-ZNF Gene Clusters between Populations

Out of all DNA-binding TF classes, KRAB-ZNF genes carry the highest number of highly genetically differentiated nonsynonymous SNPs affecting sequences in protein domains (*F*_ST_ > 0.15) (supplementary Methods.pdf, supplementary fig. S3, and supplementary table S6, Supplementary Material online). A main characteristic of the KRAB-ZNF class is that most of its members are colocated in genomic regions (gene clusters) that span more than 150 kb (Huntley et al. 2006). We thus analyzed all major KRAB-ZNF gene clusters for putative adaptive variation, where only polymorphic sites in CEU, CHB, and YRI populations with minor allele frequency >0.001 were considered. We then manually explored where in the proteins putatively selected nonsynonymous changes are located to deduce possible functional effects.

Changes in the cysteine and histidine residues involved in forming the fingerlike structure for DNA-binding of C2H2 and KRAB-ZNF proteins, in the positions −1, 2, 3, and 6 that directly interact with specific nucleotides, or in the linkers determining the space between fingers, are expected to alter the DNA-binding specificity of ZNF proteins (Ryan and Darby 1998; Laity et al. 2000; Wolfe et al. 2000). We found 42 nonsynonymous SNPs with high to very high genetic differentiation (*F*_ST_ > 0.15 < 0.95) within 11 KRAB-ZNF clusters in chromosomes 1, 3, 9, 12, 16, 18, and 19. Thirty-two of these 42 SNPs are in C2H2 genes (16 KRAB-ZNF and one non-KRAB-ZNF genes) (supplementary table S7, Supplementary Material online). Within this set of 42 nonsynonymous SNPs, 11 SNPs alter amino acid residues of ZNFs for eight KRAB-ZNF genes (*ZNF695*, *ZKSCAN7*, *ZNF502*, *ZNF571*, *ZNF132*, *ZNF10*, *ZNF90*, and *ZNF568*), whereas seven other SNPs affect amino acids of the linkers between ZNFs for four KRAB-ZNFs (*ZNF668*, *ZNF646*, *ZNF844*, and *ZNF492*), and five SNPs change the KRAB domains of three proteins (*ZNF695*, *ZKSCAN7*, and *ZNF48*) (supplementary table S7, Supplementary Material online). One highly differentiated nonsynonymous SNP modifies the amino acid residue of one of the two zinc-coordinating cysteines. Because these two cysteines (C2) together with two histidines (H2) tetrahedrally coordinate a zinc ion, which is essential for maintaining the stability and globular structure of the ZNFs (Eun 1996; Wolfe et al. 2000), this modification very likely disrupts the DNA-binding capacity of the seventh ZNF motif of ZNF492. We additionally identified seven SNPs that affect the amino acid sequence of seven C2H2 proteins outside the above mentioned functional domains (*ZNF695*, *ZNF445*, *ZNF35*, *ZNF501*, *ZNF768*, *ZNF510*, and *ZNF568*). In addition to nonsynonymous SNPs affecting KRAB-ZNF genes, nine further highly differentiated nonsynonymous SNPs occur in seven other genes located within the KRAB-ZNF clusters, of which one, *KAT8*, is a GRF (supplementary table S7, Supplementary Material online).

Taken together, some non-KRAB C2H2 and KRAB-ZNF genes carry more than one highly differentiated nonsynonymous SNP modifying the sequence of the proteins. Based on the locations of these nonsynonymous SNPs, we suggest that they influence the interaction of these ZNF proteins with DNA (changes in the fingers), RNA, or with KAP1, a cofactor that interacts with the KRAB domain promoting the repressor activity of KRAB-ZNF proteins.

### KRAB-ZNF Gene Clusters Contain Multiple Loci as Candidate Targets of Selection in CEU and CHB

Positive selection alters the allele frequencies of SNPs in the neighborhood of the selected allele, thus creating a measurable biased pattern of genetic variation. Our results suggest that at least 32 out of 85 KRAB-ZNF gene clusters of the human genome carry genes with patterns of variation consistent with positive selection in at least one of the three populations studied here (supplementary table S8, Supplementary Material online). To asses if the patterns of variation we found resemble scenarios of selective sweeps, and to evaluate whether the observed scores calculated from the empirical data are unusually high when compared with expectations under neutrality, we performed coalescent simulations using a fine-tuned (best-fit) model for human demography generated for these three human populations (Schaffner et al. 2005) and adjusting it according to Pybus et al. (2015) (see Methods).

**Fig. 2.**
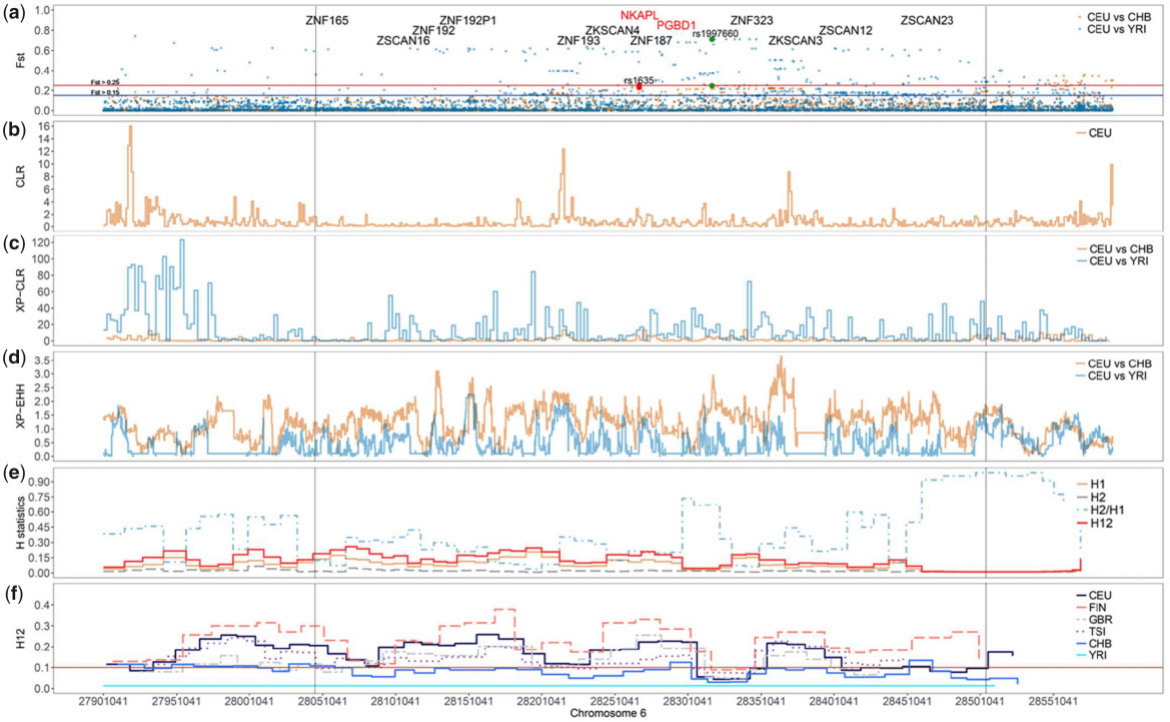
—KRAB-ZNF gene cluster located on the chromosome 6 of four European population (6: 28.04–28.42) exhibiting very high genetic differentiation (*a*), high CLR (*b*) and XP-CLR (*c*) scores, long EHH (*e*, *f*) and multiple high frequency haplotypes. Note that the scale on the *y* axis differs between plots. All values correspond to the raw scores obtained for each method. In the *F*_ST_ track (*a*), SNPs over the solid lines indicating moderate (*F*_ST_ > 0.15, blue line) and high (*F*_ST_ > 0.25, red line) genetic differentiation. Bigger dots indicate two highly differentiated SNPs, rs1635 (CEU vs. CHB, red) and rs1997660 (CEU vs. YRI, green). H12 track statistics (*e*) shows the H scores for: homozygosity of the most frequent haplotype (H1), homozygosity calculated using all, except the most frequent haplotype (H2), the ratio between H2/H1, and the combination of the most and second most frequent haplotypes (H12). In H12 track (*f*) for four populations with European, one with Asian and one with African background. The H12 threshold we defined genome wide (solid red line, 0.1). Dotted vertical line indicate extension of positively selected region within this KRAB-ZNF cluster.

Our results suggest that 15 out of the 32 KRAB-ZNF clusters mentioned above harbor at least 27 regions with patterns of variation that are unlikely to be expected under neutrality (*P* < 0.01) (table 4). Nineteen of these regions show rank scores that are among the top 1% of the whole genomic distribution. Using the H12 test (Garud et al. 2015) (supplementary Methods.pdf, Supplementary Material online), we also found that 24 of these 27 regions exhibit one or more high frequency haplotypes with EHH (from 50 kb up to 385 kb). The patterns of genetic variation in these 27 regions may have resulted from positive selection (fig. 2, supplementary figs. S4–S6, Supplementary Material online, table 4, and supplementary table S9, Supplementary Material online).

**Table 4:** KRAB-ZNF Clusters Exhibiting One to Multiple Regions Candidate for Positive Selection in Three Human Populations (CEU, CHB, and YRI)

Chromosome	Start	End	Length Haplotype	Population	GRF Genes	Non-GRF Genes	*P*
chr19	9746367	9886927	0.14	CEU	*ZNF562, ZNF812, ZNF846*		0.001
chr19	9679258	9871747	0.19	CHB	*ZNF561, ZNF812, ZNF121, ZNF562, ZNF846*		0.001
chr19	9623427	9710798	0.09	CEU	*ZNF121, ZNF426*	*OR7D2*	0.001
chr19	9433260	9579560	0.15	CHB	*ZNF177, ZNF266, ZNF560, ZNF559, ZNF559-ZNF177*		0.039
chr7	99049790	99226981	0.18	CEU	*CPSF4, ZKSCAN5, ZNF394, ZNF655, ZNF789, ZSCAN25*	*ATP5J2, FAM200A, LOC100289187, TRNA_Trp, ATP5J2-PTCD1*	0.001
chr19	12290691	12477728	0.19	CEU	*ZNF442, ZNF44, ZNF563, ZNF136*	*AK023304, AX721123*	0.001
chr19	11569316	11654956	0.09	CEU	*ZNF653*	*ECSIT, ELAVL3, CNN1*	0.001
chr19	11569316	11651077	0.08	CHB	*ZNF653*	*ECSIT, ELAVL3, CNN1*	0.001
chr19	11681367	11763981	0.08	CHB	*ZNF627, ZNF833P*	*ACP5*	0.001
chr19	11911546	12194995	0.28	CHB	*ZNF433, ZNF439, ZNF440, ZNF69, ZNF700, ZNF763, ZNF844, ZNF878, ZNF491*	*AX747405*	0.001
chr19	19518253	19658472	0.14	CEU	*NDUFA13, GATAD2A*	*CILP2, TSSK6, YJEFN3*	0.041
chr19	20219280	20473261	0.25	CEU	*ZNF90, ZNF486, ZNF826P*		0.001
chr19	22736627	22847686	0.11	CEU	*ZNF492*	*LOC440518 (GOLGA2P9), AC011516.2*	0.001
chr19	22849806	23075779	0.23	CEU	*ZNF492, ZNF723+NP, ZNF99*		0.001
chr19	22736073	22789623	0.05	CHB		*LOC440518 (GOLGA2P9)*	0.032
chr19	22797143	23066423	0.27	CHB	*ZNF492, ZNF723+NP, ZNF99*	*AC011516.2*	0.008
chr19	23167970	23274391	0.11	CEU	*ZNF728*		0.001
chr19	23566484	23647327	0.08	CEU	*ZNF91*	*LINC01224*	0.014
chr19	24159713	24258543	0.1	CEU	*ZNF254*	*AK092080, AK092150*	0.001
chr19	24165702	24249831	0.08	CHB	*ZNF254*	*AK092080, AK092150*	0.001
chr19	20912174	21159445	0.25	CHB	*ZNF85, ZNF66*		0.009
chr19	20961835	21046198	0.08	YRI	*ZNF66*		0.009
chr19	35379737	35443530	0.06	CHB	*ZNF30*	*LINC00904, and 17 PiRNAs*	0.001
chr19	37401178	37684941	0.28	CHB	*ZNF829, ZNF585A, ZNF585B, ZNF345, ZNF568, ZNF420*		0.003
chr19	38129568	38255337	0.13	CHB	*ZNF781, ZNF607, ZFP30, ZNF573*		0.039
chr19	52350176	52471785	0.12	CHB	*ZNF577, ZNF649, ZNF613, ZNF350*	*TRNA_Lys*	0.033
chr19	52350054	52407858	0.06	CEU	*ZNF577, ZNF649, ZNF613, ZNF350*		0.005
chr19	52409615	52511217	0.1	CEU	*ZNF613, ZNF350, ZNF615*	*TRNA_Lys*	0.025
chr19	52533305	52665989	0.13	CEU	*ZNF432, ZNF841, ZNF616, ZNF836*		0.014
chr19	52995729	53064163	0.07	CEU	*ZNF578, ZNF808*		0.031
chr3	40531136	40630291	0.1	CEU	*ZNF619, ZNF620, ZNF621*		0.031
chr6	28040581	28337801	0.3	CEU	*ZSCAN12P1, ZSCAN16, ZNF187, ZNF192, ZNF192P1, ZNF389, ZNF193, ZKSCAN4, ZKSCAN3, ZNF165, ZNF323, PGBD1, NKAPL*	*TRNA_Ser, TOB2P1, piRNA(DQ581281)*	0.001
chr6	28342884	28426378	0.08	CEU	*ZSCAN12, ZSCAN23*		0.003
chr12	1.33E+08	1.34E+08	0.3	CHB	*ZNF891+N, ZNF605, ZNF26, ZNF84, ZNF140, ZNF10, ZNF268*		0.001
chr1	2.47E+08	2.47E+08	0.1		*ZNF124, ZNF669, ZNF670, ZNF670-ZNF695, ZNF695*	*C1orf229*	0.001
chr3	44554702	44742478	0.19	CHB	*ZNF167, ZNF197, ZNF35, ZNF445, ZNF660, ZNF852*		0.001
chr16	31009588	31165239	0.16		*FBXL19, KAT8, SETD1A, SRCAP, TBC1D10B, ZNF48, ZNF629, ZNF646, ZNF668, ZNF688, ZNF689, ZNF747, ZNF764, ZNF768, ZNF771*7	*AK056973, BC039500, BC073928, BCKDK, BCL7C, C16orf93, CD2BP2, CTF1, DCTPP1, FBRS, HSD3B7, ITGAL, MIR4518, MIR4519, MIR762, MYLPF, ORAI3, PHKG2, PRR14, PRSS36, PRSS53, PRSS8, RNF40, SEPHS2, SEPT1, SNORA30, STX1B, STX4, VKORC1*	0.001

Note.—The patterns of variation are considered unlikely to be expected under neutrality based on the results from our simulated data. Regions found in two populations were kept separately. The significance was assessed by simulating a null model using coalescence (see Materials and Methods). An extended version of this table can be found in *supplementary table S9*, *Supplementary Material* online.

Using a curated comprehensive catalog of genes and variants associated with human diseases and the gene-disease associations from the MEDLINE database, both available in DisGeNET (Piñero et al. 2017), we identified that out of the 27 regions with one or more haplotypes at high frequency, 17 carry GRF genes that have been associated with medical conditions such as muscle weakness, obesity, hyperparathyroidism, degenerative polyarthritis, heart diseases, azoospermia, cognitive disabilities, and multiple types of cancers, among others (supplementary table S9, Supplementary Material online). Acknowledging that the biological, molecular and functional characterization, apart from their putative gene regulatory function, of most GRFs and especially of KRAB-ZNF proteins, is still incipient, we present and discuss here three regions for which functions and medical relevance has been described during the last two decades. We further discuss their putative regulatory roles in the evolution and adaptation of human-specific traits. Extended information for other regions can be found in the supplementary table S10, Supplementary Material online, and in the supplementary figs. S4–S6, Supplementary Material online.

### Selective Sweep on a KRAB-ZNF Gene Cluster in CEU Is Associated with Schizophrenia in CHB

A KRAB-ZNF cluster located on the chromosome 6 (28.04–28.42) displays multiple regions with high CLR, XP-CLR, and XP-EHH scores in CEU. It also contains variants that suggest very high genetic differentiation (*F*_ST_) between CEU compared with CHB and YRI (fig. 2). In addition, more than 100 SNPs exhibit very high *F*_ST_ (>0.15 < 0.52) when comparing CEU with the other two populations (fig. 2, *F*_ST_ track). We also detected that the level of genetic differentiation in this region was unusually high when compared with regions evolving under neutrality scenarios (simulated data, *P* < 0.001, supplementary fig. S7, Supplementary Material online). Genetic variation within this KRAB-ZNF gene cluster has been associated with at least three medical conditions in humans: hemochromatosis (iron overload, the most prevalent genetic condition in Europeans), CD4:CD8 lymphocyte ratios, low production of CD8+ effector memory (T_EM_) and double negative (T_DN_) T-cells, and schizophrenia.

**Fig. 3.**
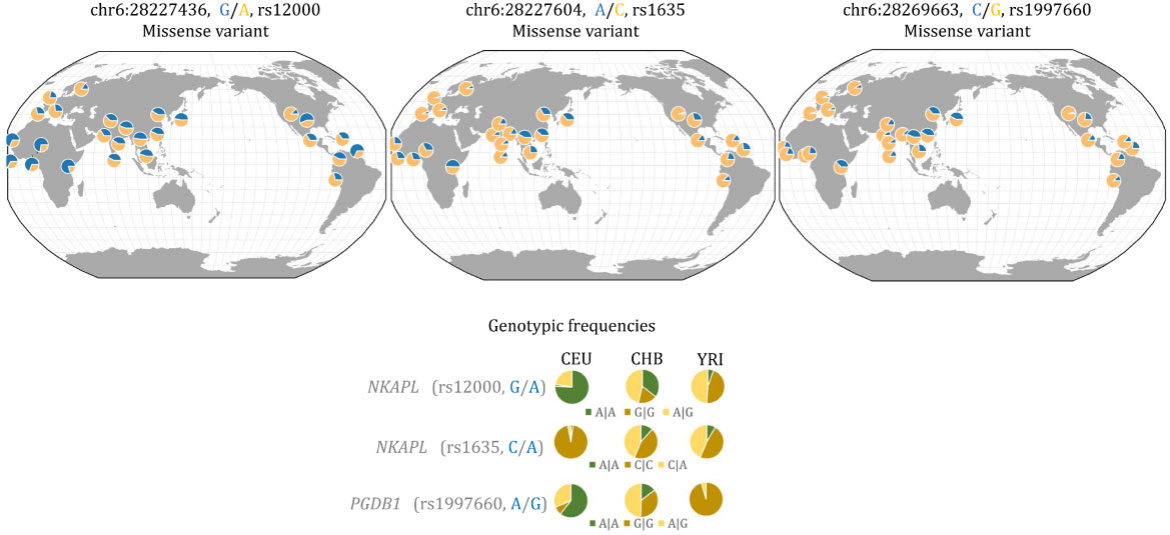
—Three missense variants located in two genes within a KRAB-ZNF gene cluster that might have undergone positive selection in European populations. Top left and middle, allelic frequencies of two nonsynonymous SNPs located in *NKAPL* gene. Top right, allelic frequencies of one nonsynonymous SNP located in *PGDB1* gene. Bottom, genotypic frequencies for CEU, CHB, and YRI.

This KRAB-ZNF cluster is composed of at least 16 genomic elements: 11 GRF genes (*ZSCAN16*, *ZNF187*, *ZNF192*, *ZNF389*, *ZNF193*, *ZKSCAN4*, *ZKSCAN3*, *ZNF165*, *ZNF323*, *PGBD1*, and *NKAPL*), three pseudogenes (*ZSCAN12P1*, *ZNF192P1*, and *TOB2P1*), one tRNA-Ser and one piRNA-DQ581281. Out of the 100 highly differentiated SNPs, only three correspond to nonsynonymous SNPs in coding regions of two genes, namely in the first exon of *NKAPL* (rs12000 and rs1635) and in the seventh exon of *PGBD1* (rs1997660) (fig. 2, *F*_ST_ track, supplementary table S10, Supplementary Material online). Interestingly, the rs1635 genotype is almost fixed in CEU with about 95% of the individuals carrying the variant *C|C*. In contrast, this genotype is found in only about 50% of the individuals from CHB and YRI. In these populations, the heterozygous state *A|C* is frequent with 40% and 45%, respectively (fig. 3). Allelic variants in rs1635 and rs12000 have been associated with schizophrenia in Han Chinese (Chen et al. 2014; Wang et al. 2015). Conversely to rs1635, the genotype *G|G* for SNP rs1997660 (*PGBD1* gene) is found in almost all individuals from YRI (95%), whereas it is at lower frequency in CHB (36%) and at very low frequency in CEU (0.8%) (fig. 3). Considering the clear differences in genotype frequencies for rs1635 and rs1997660 between populations, we suggest that these two SNPs are involved in local adaptation.

Further exploration suggests that genes within this KRAB-ZNF cluster might have experienced a recent selective sweep in CEU and in four other European populations: Finnish in Finland, Toscani in Italia, and British in England and Scotland (fig. 2, H12 track). By using H12 statistics, we were able to detect that this KRAB-ZNF cluster contains two regions with one predominant haplotype with long EHH about five kb apart from each other that together span about 400 kb in CEU (fig. 2 and supplementary fig. S3*h* and *i*, supplementary table S10, Supplementary Material online). The length of the EHH for these European populations ranges from 300 kb in Italian to 490 kb in Finnish (supplementary fig. S8, Supplementary Material online), suggesting that it is very likely that the extension, strength and time of this selective event differs across European populations.

### Positive Selection on a Human-Specific KRAB-ZNF Cluster Involved in Eye Development

Two closely located regions on chromosome 19, 7 kb apart from each other, encompassing together around 340 kb (chr19: 22.7–23.04), carry very high genetic differentiation (*F*_ST_) and long EHH in CEU and CHB populations. When compared with YRI population, these regions also showed high CLR and XP-EHH scores, which suggests a selective sweep (supplementary fig. S9, Supplementary Material online). By analyzing the ratio from the H2/H1statistics, which tends to increase as the sweep becomes softer (Garud et al. 2015), we suggest that CEU may have experienced a harder sweep than CHB population (supplementary fig. S4*A*, *d* and *e* and supplementary fig. S9, Supplementary Material online). Indeed, it is possible to observe two well defined haplotypes that are at high frequency for CHB (supplementary fig. S4*B*, *e* and *f*, Supplementary Material online). One gene within this region, the human-specific gene *ZNF492* (Nowick et al. 2010), was recently reported as a positively selected gene in nine individuals with European background (Huber et al. 2016).

ZNF492 regulates the expression of the retinal pigment epithelium (RPE) specific protein (RPE65) (Lu et al. 2006). We found that both genes, *ZNF492* and *RPE65*, display higher average expression in the human retina compared with most other tissues. *ZNF492* is more highly expressed in the RPE during development than during adulthood (supplementary fig. S10, Supplementary Material online), suggesting that it might play an important role during RPE development. Three nonsynonymous SNPs can be found within *ZNF492*. Two missense SNPs (rs138844698 and rs141989264) concern amino acid changes that are fully conserved in CEU and CHB (100%), but not in YRI (66%). These two variants alter the amino acid configuration of the linker between the first and second ZNFs of *ZNF492*, which is expected to alter the cap configuration of the C terminus (C-cap) of the first finger, tweak its DNA-binding affinity (Laity et al. 2000; Wolfe et al. 2000), and potentially affect its regulatory properties. The third SNP (rs144581197) changes a highly conserved cysteine residue (TGT) with essential role in the proper folding of the seventh ZNF of ZNF492 to a tyrosine (TAT) (supplementary fig. S11, Supplementary Material online). Although the *A* allele for rs144581197 is relatively frequent in YRI (∼56%) and other African populations (between 39% and 57%, of the African populations from the 1000 genomes project), it is found in <1% of individuals from CEU and CHB (supplementary fig. S11, Supplementary Material online). The *A* allele is also present in individuals from populations located in regions with moderate to high solar UV index (WHO 2002): Puerto Ricans, 8%; Colombians, 7%; Iberian Spaniards, 4%; Peruvians, 3.5%; Mexicans, 2%; Tuscans, 2%, which is intriguing for a gene expressed in the RPE and other eye tissues.

This KRAB-ZNF region resulted from multiple intrachromosomal segmental duplications (Bailey et al. 2002) that gave origin to *ZNF492*. Although the sequence similarity of the entire region is about 98–99% between modern and archaic humans (Denisovan and Neandertal, respectively), it is only about 89% between human and chimpanzee. Therefore, it is likely that the duplication events and the rearrangements of this region occurred during the evolution of the homo species. We additionally explored the three nonsynonymous SNPs of *ZNF492* in Denisovan and Neanderthal. We conclude that individuals from archaic humans carry the variants fixed in CEU, CHB. This suggests that the alternative variants we observed for these SNPs, especially rs144581197, might have resulted from de novo mutations in YRI, and other African populations.

### Recent Incomplete Selective Sweep on a KRAB-ZNF Gene Cluster on Chromosome 3 Might Contribute to the Male Fertility

**Fig. 4.**
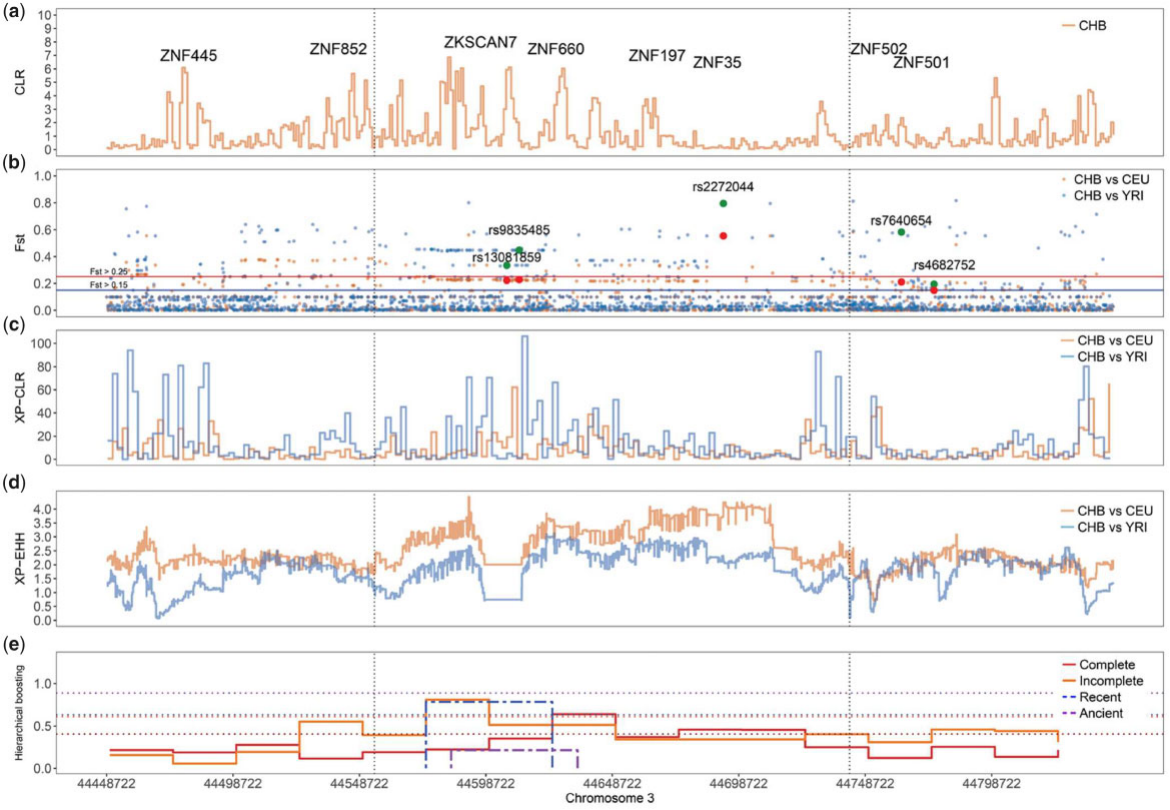
—KRAB-ZNF gene cluster exhibiting hard sweep on the chromosome 3 from CHB population (3: 44.55–44.74). Three methods for detecting positive selection and *F*_ST_ for measuring genetic differentiation produced very high scores for this region (*a*–*d*) when compared with other regions genome wide. Note that the scale on the *y* axis differs between plots. All values correspond to the raw scores obtained for each method. *F*_ST_ (*b*) and XP-EHH (*d*) results indicate very high genetic differentiation and a haplotype with EHH that spans about 188 kb (vertical dotted lines). This KRAB-ZNF cluster contains eight ZNF genes. The regions flanking up and downstream of this 188-kb haplotype also exhibit EHH, which suggests that they correspond to the same selective sweep (about 272 kb). Four highly differentiated nonsynonymous SNPs (green: CHB vs. YRI; red: CEU vs. YRI) in regions coding for protein domains of *ZKSCAN7*, *ZNF35*, *ZNF501*, and *ZNF502* may be of functional relevance. Hierarchical boosting results (*e*) suggest this correspond to an incomplete recent selective sweep. Solid and dotted horizontal lines indicate thresholds for *F*_ST_ (blue: *F*_ST_ > 0.15, red: *F*_ST_ >0.25) and boosting significance thresholds as defined by Pybus et al (2015) (red: complete, orange: incomplete, blue: recent, and purple: ancient), respectively.

One KRAB-ZNF cluster located on chromosome 3 (44.55–44.74) exhibits long EHH, high scores for CLR and XP-CLR, and high genetic differentiation between CHB and the other two populations (fig. 4). The EHH spans about 272 kb in a region that contains three KRAB-ZNF genes (*ZNF167*, *ZNF197*, and *ZNF445*) and three C2H2 (*ZNF35*, *ZNF660*, and *ZNF852*). The hierarchical boosting data for classifying hard sweeps in human populations (Pybus et al. 2015) indicated that this KRAB-ZNF cluster might have experienced an incomplete and recent selective sweep in CHB (fig. 4).

Further exploration revealed six nonsynonymous SNPs with high genetic differentiation across populations. The SNP rs2272044, located in the second exon of the gene *ZNF35*, exhibit great genetic differentiation (*F*_ST_ > 0.56 < 0.8) (fig. 4). Although about 98% of the individuals from CHB carry the allele G, this is only present in 30% of the individuals from CEU, and absent in YRI (100%). Despite the understanding of functional roles of *ZNF35* gene in humans is still scarce, its highly conserved homolog in mice suggests that it might play important roles in male fertility (Przyborski et al. 1998) by regulating pathways involved in the release of differentiated spermatogenic cells into the central lumen of the seminiferous tubule in testis from adults (Zhou et al. 2010). The deletion of *ZNF35* causes the premature release of undifferentiated pachytene spermatocytes into the lumen in subfertile individuals (Zhou et al. 2010). Certainly, *ZNF35* is highly expressed in pachytene spermatocytes and round spermatids (Przyborski et al. 1998; Zhou et al. 2010), as well as in testis (GTEx Consortium et al. 2017), and its protein has been found in spermatogenic cells (Zhou et al. 2010).

Two other nonsynonymous SNPs located in the gene *ZKSCAN7* (rs13081859, rs9835485) also suggest very high genetic differentiation (*F*_ST_ > 0.23) *F*_ST_ (fig. 4 and supplementary table S7, Supplementary Material online). These two SNPs affect the amino acid sequence of the KRAB domain and the first amino acid between the two histidines of the first ZNF of *ZKSCAN7*, respectively. Both variants may have functional implication in the interactions of ZKSCAN7 with the coregulator *KAP1* (*TRIM28*) and the DNA-binding sites.

## Discussion

We generated an up-to-date catalog for human GRFs, comprising 3,344 protein-coding genes, to investigate if these types of genes are frequently observed in regions with signatures of positive selection among human populations. Although about 53% of the GRFs located in candidate regions for selection for YRI seem to be population specific, the greater overlap detected between CEU and CHB suggests that genetic variation in the majority of the GRFs for CEU and CHB has resulted from evolutionary processes that followed the migration Out of Africa. At least five GRF classes are overrepresented in candidate regions for positive selection in three human populations (CEU, CHB, and YRI), pointing to an enormous potential for regulatory diversity between populations. We also detected that at least 15 KRAB-ZNF gene clusters harbor loci with high genetic differentiation between pairs of populations, considerable reduction in genetic variation within populations, and EHH that spans between 50 and 385 kb. Our results are unlikely to be confounded by demographic events, for instance, bottlenecks or population expansions, because we implemented several steps that are in agreement with recommended practices when identifying candidate regions for positive selection, among them our outlier approach, analyzing the site frequency spectrum, using haplotype based methods and coalescent simulations for neutrality (Wollstein and Stephan 2015). Our results strongly indicated that the genetic variation we characterized here may be responsible of subtle to rough regulatory changes of relevance for human adaptive regulatory diversity.

### Positive Selection of C2H2 Genes as a Potential Source for Regulatory Diversity

By far the highest incidences of genes within candidate regions for positive selection was found for KRAB-ZNF genes. This class has also experienced lineage-specific duplications and functional divergence in primates (Nowick et al. 2010, 2013; Najafabadi et al. 2015) and has been implicated in speciation processes (Emerson and Thomas 2009; Nowick et al. 2013) and in the suppression of endogenous retrovirus (ERVs) (Thomas and Schneider 2011; Jacobs et al. 2014; Lukic et al. 2014; Santoni de Sio 2014). Therefore, it becomes interesting that genes from KRAB-ZNF class show very high levels of genetic differentiation at population-specific level in humans.

The ways in which KRAB-ZNF and other C2H2 proteins recognize and bind to DNA to control the expression of other genes have been documented in considerable detail (Wolfe et al. 2000), allowing it to draw conclusions about functional impacts of the genetic variation we observed for KRAB-ZNF genes. KRAB-ZNF proteins typically contain modular Cys2-His2-ZNF finger domains joined together in tandem arrays by linker regions (Huntley et al. 2006). Changes in the cysteine or histidine residues involved in forming the fingerlike structure, in the positions −1, 2, 3, and 6 that are binding specifically to DNA, or in the linkers between the finger domains can alter the regulatory specificity of KRAB-ZNF proteins (Laity et al. 2000; Wolfe et al. 2000). Such changes can broadly affect the phenotype, for instance, a mutation in the linker between the 18th and 19th C2H2 finger domain of *ZNF407* (the c.C5054G/p.S1685W) causes cognitive impairment (Kambouris et al. 2014). We identified nonsynonymous SNPs in functionally relevant positions of ZNF proteins with very high genetic differentiation across human populations, even though most of this variation lays outside the sequences coding for protein domains of KRAB-ZNF. This is consistent with a previous study that suggested that nonsynonymous SNPs on protein domains and contacting residues of older KRAB-ZNF genes are prone to evolve under purifying selection, that is, are less frequent (Kapopoulou et al. 2016). The variants that can be predicted to change the functions of the KRAB-ZNF proteins should be followed up experimentally to reveal their functional consequences.

Variation in the tandem array of ZNF domains may result in diversifying mechanisms to downregulate the expression of newly evolved TEs in humans (Lukic et al. 2014). In turn, changes in expression of TEs might lead to important phenotypic differences between human populations (Wang et al. 2017). For instance *ZNF35*, a gene that carries highly differentiated nonsynonymous SNPs between the human populations, and that is located in a region we identified as strong candidate for a hard selective sweep in CHB, can bind and regulate specific classes of EREs (Najafabadi et al. 2015). Human-specific ERVs affect spermatogenesis and lead to male infertility (Prudhomme et al. 2005), a global medical condition with lower prevalence in China and other Asian countries (Nosrati et al. 2017). *ZNF35* is involved in controlling normal spermatogenesis, and ultimately male infertility in human (Zhou et al. 2010). Liu et al. (2017) recently suggested that this region has convergently evolved in six Asian populations: Buryat, CHB, Amagasaki, Tibetan, Hui, and Han. Consequently, we suggest that this region, including the variants reported here for *ZNF35*, contributes to regulatory differences across human populations that could be of relevance for male fertility.

### KRAB-ZNF Gene Clusters Have Experienced Selective Sweeps and Probably Contributed to Rapid Human Adaptation

Regions with EHH, reduced variability, low recombination rates, and high genetic differentiation across populations are indicative for positive selection (Sabeti et al. 2007; Vitti et al. 2013). We found that at least 27 regions (>50 kb) within 32 KRAB-ZNF gene clusters exhibit unusually biased patterns of genetic variation when compared with expectations under neutrality, high differentiation between populations, reduced genetic variation within populations, and EHH. Twenty-four of these regions have one or more high frequency haplotypes with EHH, suggesting that selective sweeps in these KRAB-ZNF clusters may have taken place, especially in CHB and CEU populations. Three other KRAB-ZNF clusters, on chromosomes 1, 3, and 16 of CHB seem to have experienced harder selective sweeps. The composition of genes within these 27 regions emphasizes that multiple selective sweeps, with different strength and age, might have taken place in regions rich in KRAB-ZNF genes, probably conferring regulatory adaptive responses in human populations, for instance, CHB and CEU.

Human traits and capacities have evolved as result of local adaptation to different environmental pressures. Genes that control more than one trait, as GRFs do, may be prone to antagonistic pleiotropy, where at least the regulation of one trait has a positive effect on fitness, but also negatively impact on other traits (Williams 1957; Corbett et al. 2018). Thus, functional disease association of genes located within these 27 regions might reveal likely biological effects of the positively selected variation at population-specific level. For these regions, we found associations that might explain, for instance, differences in prevalence of phenotypical alterations such as in visual impairment, immunological response, mental disorders, body weight, and multiple types of cancer.

### Selective Sweep Might Have Fine-Tuned the Regulation of CD8+ T Cell Response and Influence the Prevalence of Schizophrenia

An interesting KRAB-ZNF cluster on chromosome 6 shows signatures of a selective sweep in CEU and likely in at least four other European populations (Finnish, Italy, England, and Scotland). Previously documented genetic variation within this region has been associated with hemochromatosis, the most prevalent inherited disorder in people with northern European ancestry (Adams 2015). Hemochromatosis patients from Portugal, US America, and Norway, carrying the most frequent haplotype produce significantly fewer mature CD8+ T-cells (T_EM_ and T_DN_ CD28^−^CD27^−^) when compared with patients carrying the less frequent haplotype (Macedo et al. 2010; Costa et al. 2013). Genetic variation within this KRAB-ZNF has been also significantly associated with CD8 T-cell production in more than 2,500 individuals (Ferreira et al. 2010). One gene located in this region, *NKAPL*, is a transcriptional repressor acting on Notch target genes (Okuda et al. 2015) and is essential for T-cell development and maturation (Pajerowski et al. 2009). Loss-of-function of NKAPL results in a significant decrease of CD8+ single (T_SP_) and double positive (T_DP_) T-cells and in a significant disproportionate increase of CD8+ T_DN_ during T-cell development (Pajerowski et al. 2009). We suggest that the genomic differentiation we detected between CEU and CHB and YRI has a measurable functional effect on the maturation and development of CD8+ T-cells, significantly changing the proportions of CD8+ cells, by mainly modulating the premature maturation of T_EM_ and T_DN_ types. We speculate that a positive selective event in the *NKAPL* gene has occurred in CEU and other European populations, whereas *NKAPL* probably evolved under balancing selection (overdominance) in CHB, YRI, and other human populations. As result, individuals carrying the most frequent haplotype found for *NKAPL* in CEU will produce much less T_EM_ and T_DN_ CD8+ T-cells, whereas individuals carrying other haplotypes will be affected by a dysregulation of the CD8 T-cell development through *NKAPL*.

The SNP rs1635 in *NKAPL* has further been associated with schizophrenia in patients from the Han Chinese and Jewish populations (Yue et al. 2011; Alkelai et al. 2012; Kitazawa et al. 2012; Zhang et al. 2013; Wang et al. 2015; Owen et al. 2016). Human immune response and altered T-cell immunity have been acknowledged to be associated with schizophrenia, although, the connection has not been fully depicted yet. Interestingly, T_EM_ and T_DN_ T-cells, which development and maturation is altered by *NKAPL* (see above), are specifically connected with the immune surveillance of the central nervous system in humans (Smolders et al. 2013; Korn and Kallies 2017). We speculate that genetic variation in *NKAPL* gene causes a disbalance in CD8+ T_EM_ and T_DN_ production and might be one of the reasons for the higher prevalence of schizophrenia in populations with Asian background when compared with Europeans. Our conclusions support findings from Fujito et al. (2018), who depicted how evolutionary processes such as admixture and environmental stressors may have influenced particular genetic variants in Asian populations.

In addition, CD8+ T_EM_ and T_DN_ T-cells specially produce granzyme K in the human brain (Smolders et al. 2013), a serine protease found in cytotoxic lymphocytes. It is possible that granzyme K promotes inflammation, cytokine production (Wensink et al. 2015), and performs other functions that can alter the neurological processes in the brain. This scenario is in line with the results obtained by Wu et al. (2017), who suggested that *NKAPL* rs1635 (T152N mutation) may be related to central nervous system development and significantly associated with the plasma levels of Intercellular adhesion molecule–1 (sICAM1), an important cell adhesion molecule with essential roles in inflammatory processes.

Taken together, we contemplate a scenario in which individuals carrying the less frequent variants for the SNP rs1635, or other SNPs that lead to gain-, switch-, or loss-of-function in NKAPL, may produce drastic changes in CD8+ T_EM_ and T_DN_ amounts, which might result in higher presence of these T-cells in their brains, and probably higher amounts of granzyme K. Although a full characterization of the impact of *NKAPL* diversity on the development of CD8+ T-cells and central nervous system functions has yet to be performed, our results provide a testable hypothesis for the molecular basis that might underlie differences in the prevalence and risk factors for schizophrenia between human populations.

### Selective Sweep in CEU and CHB Might Be Associated with Regulatory Pathways of the Retinal Epithelium

We identified a KRAB-ZNF cluster of about 340-kb EHH on chromosome 19 of CEU and CHB populations. The genomic architecture of this KRAB-ZNF cluster resulted from multiple intrachromosomal duplications (Bailey et al. 2001, 2002) that gave origin to the genes *ZNF492* and *ZNF99*. Based on the sequence similarity we found between AMHs, Neandertal and Denisovan, we suggest that this region evolved before AMHs and archaic humans split from their last common ancestor. This region likely underwent positive selection in at least two human populations, CEU and CHB. Similar results were found for *ZNF492* in nine unrelated individuals with European background (Huber et al. 2016).

RPE65, a highly tissue-specific protein (Lu et al. 2006), is essential in the visual cycle pathway that regenerates the chromophore 11-*cis*-retinal (rhodopsin). Rhodopsin protects the retina and choroid against light rays (Hu et al. 2008). *ZNF492* enhances the expression of *RPE65* (Boulanger et al. 2000; Lu et al. 2006), an essential protein for RPE development (Lu et al. 2006). *RPE65* is mostly expressed until late development of the retinal epithelium, 1–4 days after birth, and 2 days before the maturation of the photoreceptor cells (Lu et al. 2006). Both genes, *ZNF492* and *RPE65* seem to have very similar expression patterns in the human RPE and retina (Bryan et al. 2018), although the fold-change ratios between *RPE65* and *ZNF492* of adult humans show very different patterns when compared with other retinal tissues and cell lines. Considering that ZNF492 coregulates and enhances the expression of *RPE65* gene in RPE cells in humans (Boulanger et al. 2000; Lu et al. 2006), we hypothesize that this KRAB-ZNF region might confer variation that influences the development of the human eye, for instance, the retinal epithelium. Supporting our hypothesis, genetic variants within this KRAB-ZNF cluster has been functionally associated with pathologies of the human eye in Caucasians (Li et al. 2012).

We found three missense variants in *ZNF492* that are expected to alter the regulatory properties of ZNF492 in humans and thus, might alter the expression of *RPE65* and other target genes. The SNP (rs144581197) results in a change of a cysteine to a tryptophan residue (C314Y), rendering the seventh ZNF domain unfunctional in more than half of the individuals from YRI. Interestingly, also between 39% and 57% of the individuals from the seven other African populations (1000 Genomes Project Consortium 2012) carry this mutation. Further, this variation can also be found in individuals from populations located in latitudes with moderate to high sun rays exposure (WHO 2002), although with lower frequency than in the African populations. The other two SNPs (rs138844698 and rs141989264) modify the C-cap conformation of the linker between the first and second finger of ZNF492, thus likely tweaking its DNA-binding affinity (Laity et al. 2000; Wolfe et al. 2000) and regulatory functions. Given that these variants exist almost not at all in CHB and CEU, we suggest that population-specific variation of *ZNF492* may alter the development of RPE during early human stages, and posteriorly influence the regeneration of rhodopsin during adulthood and senescence in humans, presumably in adaptation to different exposure to sun rays. UV and visible radiation can damage the RPE and other structures in the eye causing different medical conditions; for instance, macular degeneration, which has a higher prevalence in Caucasian populations (Noell et al. 1966; Margrain et al. 2005; Delcourt et al. 2014).

## Conclusions

Using the most recent catalog for GRFs, the information from the 1000 genomes project, and data obtained by multiple methods for detecting positive selection in humans, we identified GRF genes located in genomic regions that may have undergone positive selection in at least one of three human populations. Our results present several scenarios where five of the largest classes of GRFs may have contributed to adaptive regulatory changes within human populations. Further inspection of nonsynonymous variants suggests how genetic variation, mainly in non-KRAB C2H2 and KRAB-ZNF ZNF classes, could confer regulatory diversity in humans, thus possibly contributing to the evolution of particular traits. Because KRAB-ZNF proteins control the expression of many genes, it is very likely that KRAB-ZNF genes located within target regions for positive selection are prone to antagonistic pleiotropy. We found that many KRAB-ZNF gene clusters targeted by natural selection have been connected with multiple medical conditions of relevance for human health, for instance, muscle weakness, obesity, hyperparathyroidism, degenerative polyarthritis, heart diseases, azoospermia, cognitive disabilities, and multiple types of cancers, among others. Our work identified several interesting candidates for further functional investigations to shed light on the evolution of human traits.

## Supplementary Material

Supplementary data are available at *Genome Biology and Evolution* online.

## Supplementary Material

evz131_Supplementary_Data
