# Supplementary material for: Genetic Variation in Human Gene Regulatory Factors Uncovers Regulatory Roles in Local Adaptation and Disease
Source: Genome Biol Evol. 2019 Jun 22;11(8):2178–93. doi: 10.1093/gbe/evz131 (PMC6685493; doi:10.1093/gbe/evz131)
Supplement: evz131_Supplementary_Data [file evz131_supplementary_data.zip › Supplementary_figures_S1-S11_Final.docx]

**Supplementary figures**

Genetic variation in human gene regulatory factors uncovers regulatory roles in local adaptation and disease

Perdomo-Sabogal, A., and Nowick, K.

**Table of Contents**

**Classification of DNA-binding TF genes included in our catalog (Figure S1).2**

**Overlap lists of GRF genes located in candidate regions for selection (Figure S2)3**

**Counts of genetically differentiated non-synonymous SNPs in regions coding for protein domains per GRFs class (Figure S3)4**

**Human KRAB-ZNF gene clusters harboring multiple high frequency haplotypes (Figure S4)5**

**Selection on KRAB-ZNF gene cluster on chromosome 1 of CHB (Figure S5)6**

**Selection on KRAB-ZNF gene cluster on the chromosome 16 of CHB (Figure S6)7**

***F_ST_* scores for genetically differentiated KRAB-ZNF in chromosome six from CEU (Figure S7)9**

**Selection on KRAB-ZNF gene cluster on chromosome six of European populations (Figure S8)10**

**Positively selected human-specific region in CEU and CHB (Genes ZNF492 and ZNF99) (Figure S9)11**

**Differential expression for *RPE65* and *ZNF492* across all tissues (Figure S10)12**

**Genetic variation introducing functional variation in two zinc fingers of *ZNF492* (Figure S11)13**

**References14**


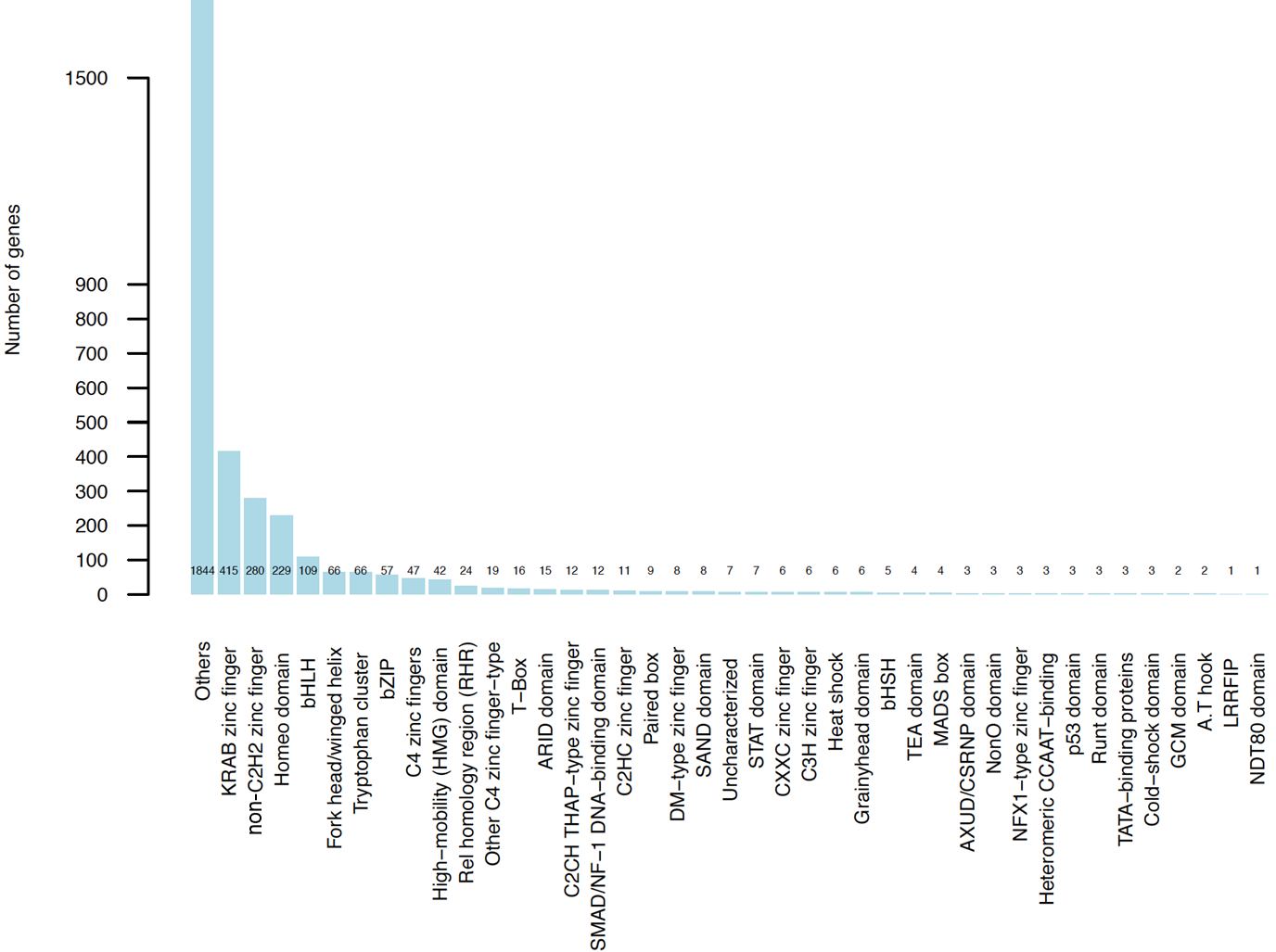


Figure S1. Classification of DNA-binding TF genes included in this work (Catalog). The grouping follows the classification generated by Wingender et al, 2015. C2H2 and KRAB-ZNF zinc fingers were separated in two different classes, based on the presence of the KRAB domain.


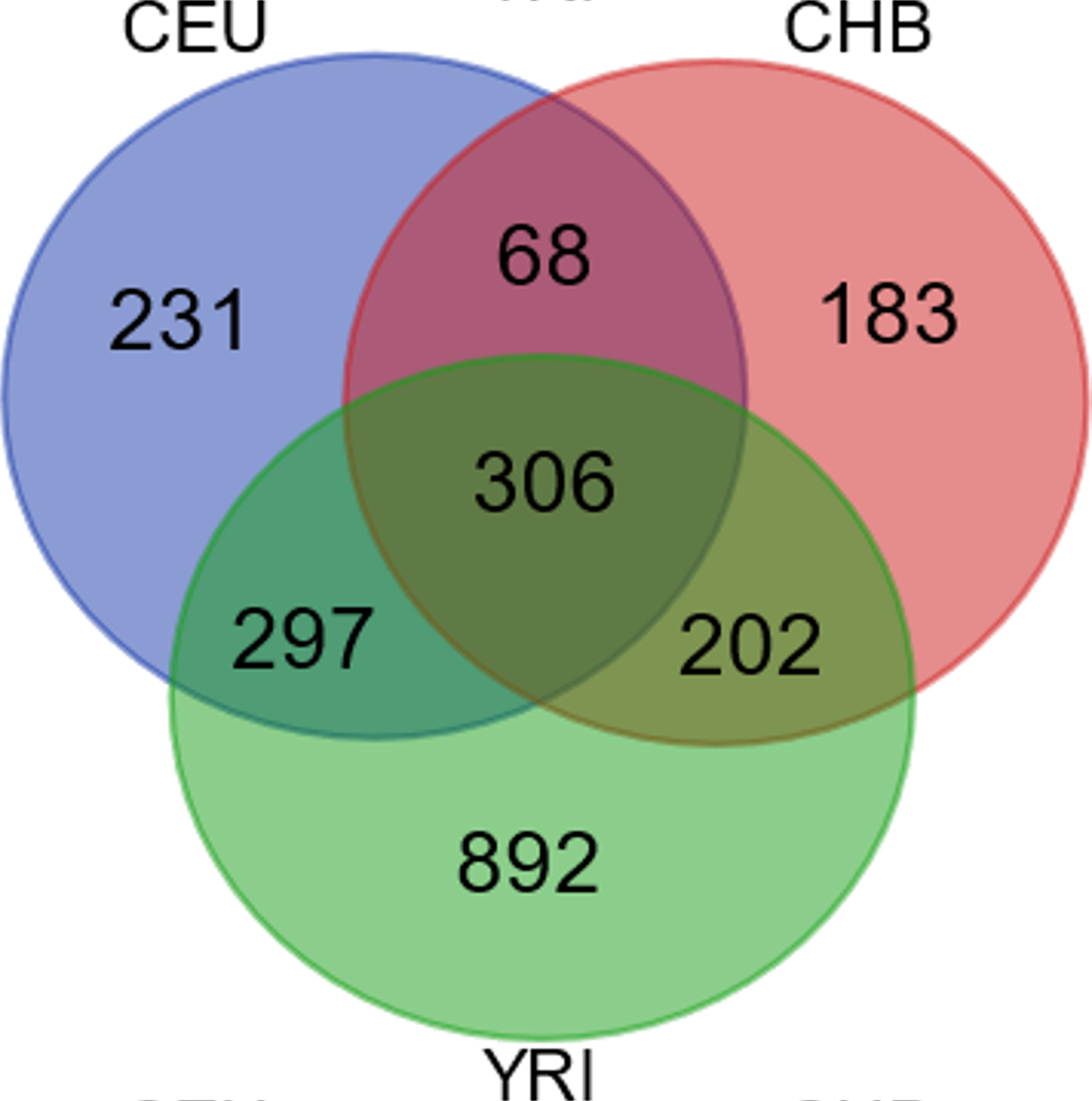


Figure S2. Overlap of GRF genes located in candidate regions with patterns of variation consistent with positive selection among three human populations: CEU (Utah residents with Northern and Western European ancestry), CHB (Han Chinese, China), YRI (Yoruba in Ibadan, Nigeria)


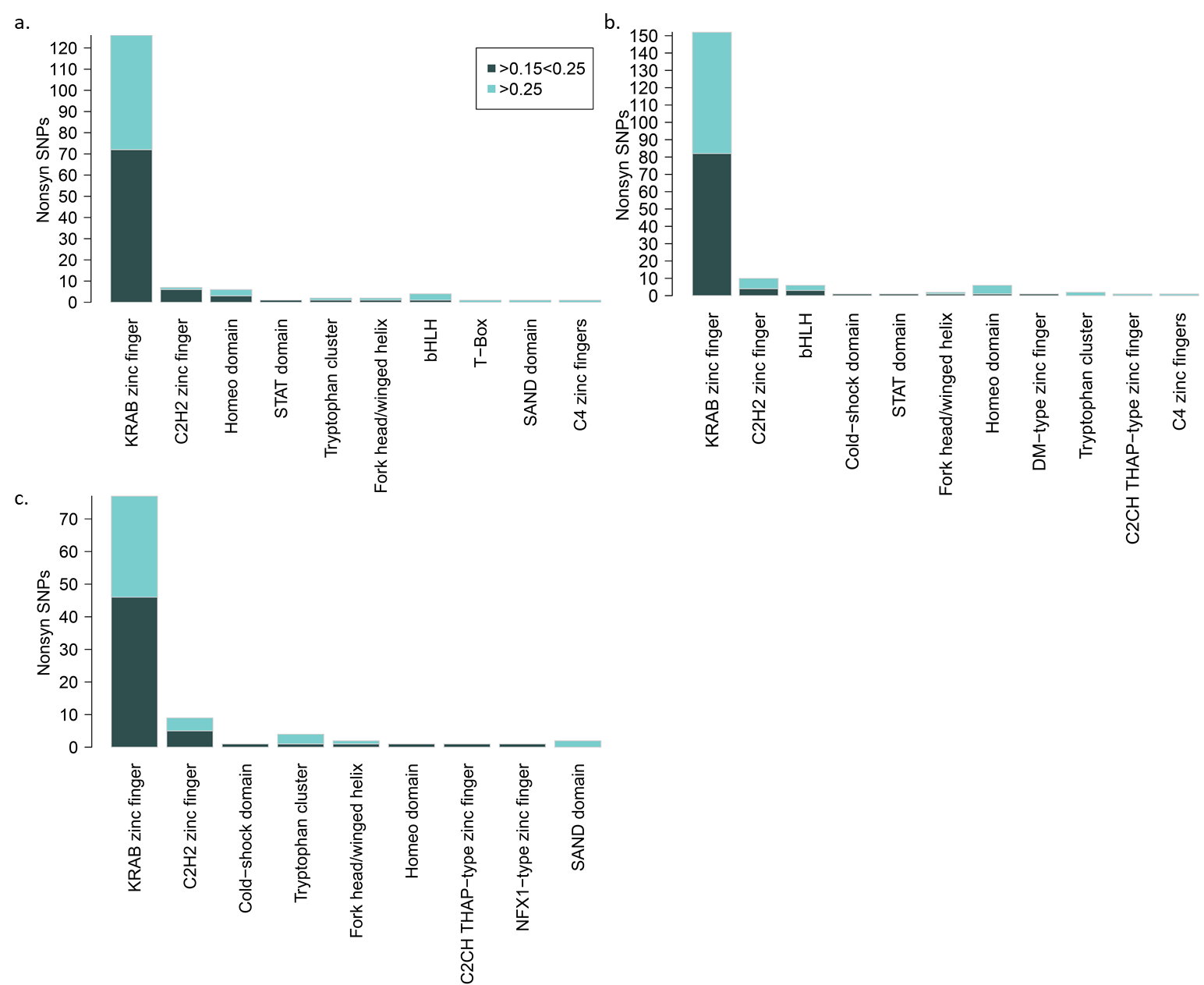


Figure S3. Total number of highly genetically differentiated non-synonymous SNPs (*F_ST_*) in regions coding for sprotein domains found for all TF genes from the 40 DNA-binding classes. **a.** CEU versus YRI. **b.** CHB versus YRI. **c.** CEU versus CHB. Notice that labels on the x and y axis are different between a, b, and c. The class C2H2 corresponds to those C2H2 zinc finger genes that do not have a KRAB domain.

##
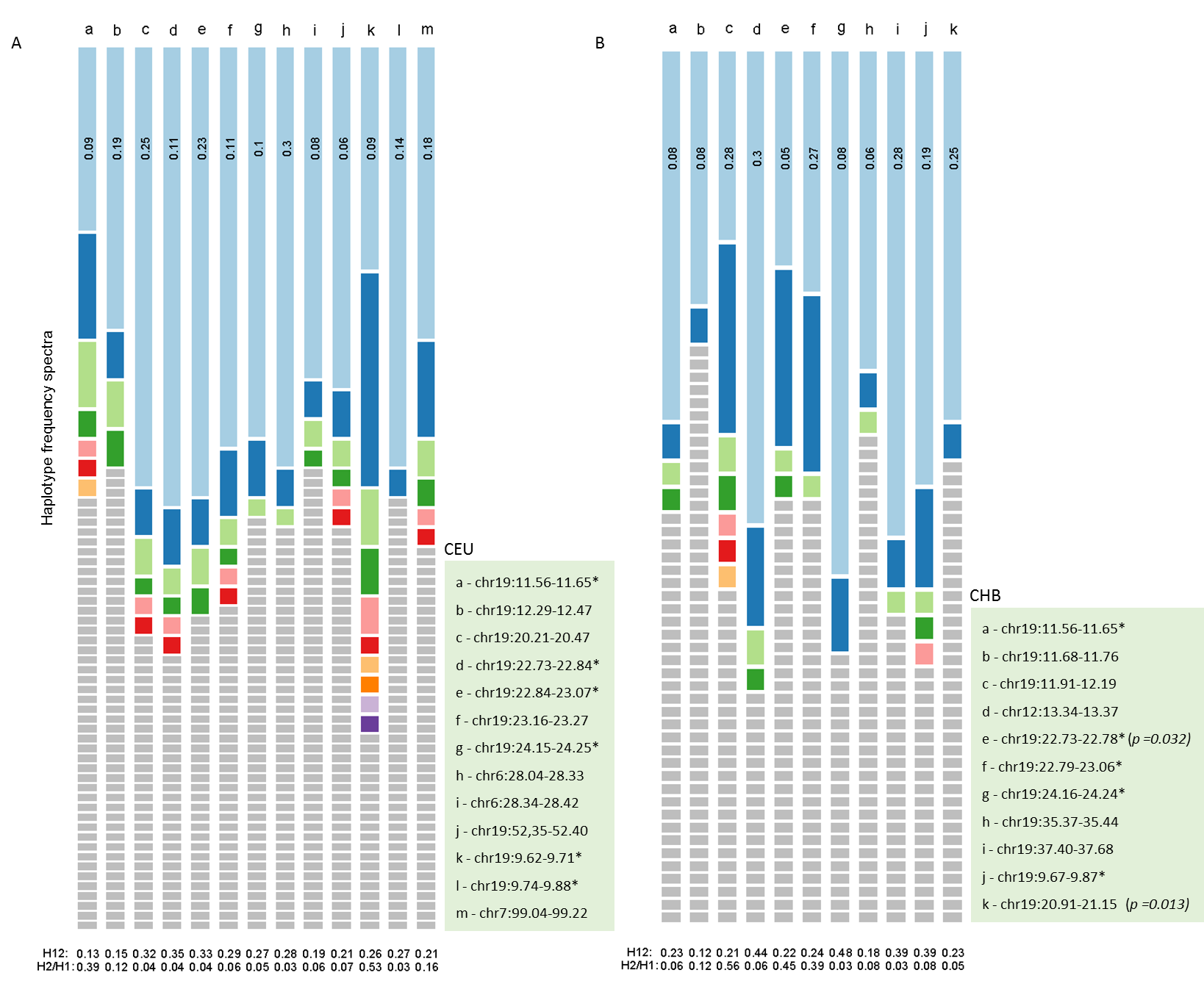


Figure S4. Regions located within human KRAB-ZNF gene clusters harboring multiple high frequency haplotypes and EHH (>50 kb) in two human populations. A. CEU. B. CHB. Each color represents one haplotype and its frequency. The length of the region exhibiting EHH is indicated by the number in the blue bars (Mbps). The length of the color represents the frequency in which each haplotype is present. Light blue color represents the most frequent haplotype. Grey color represents singletons. x axis, H2/H1 and H12 statistics. Ratios of H2/H1 increase as a selective sweep becomes softer , while high H12 scores suggest the strength of the selective sweep (Garud et al. 2015).


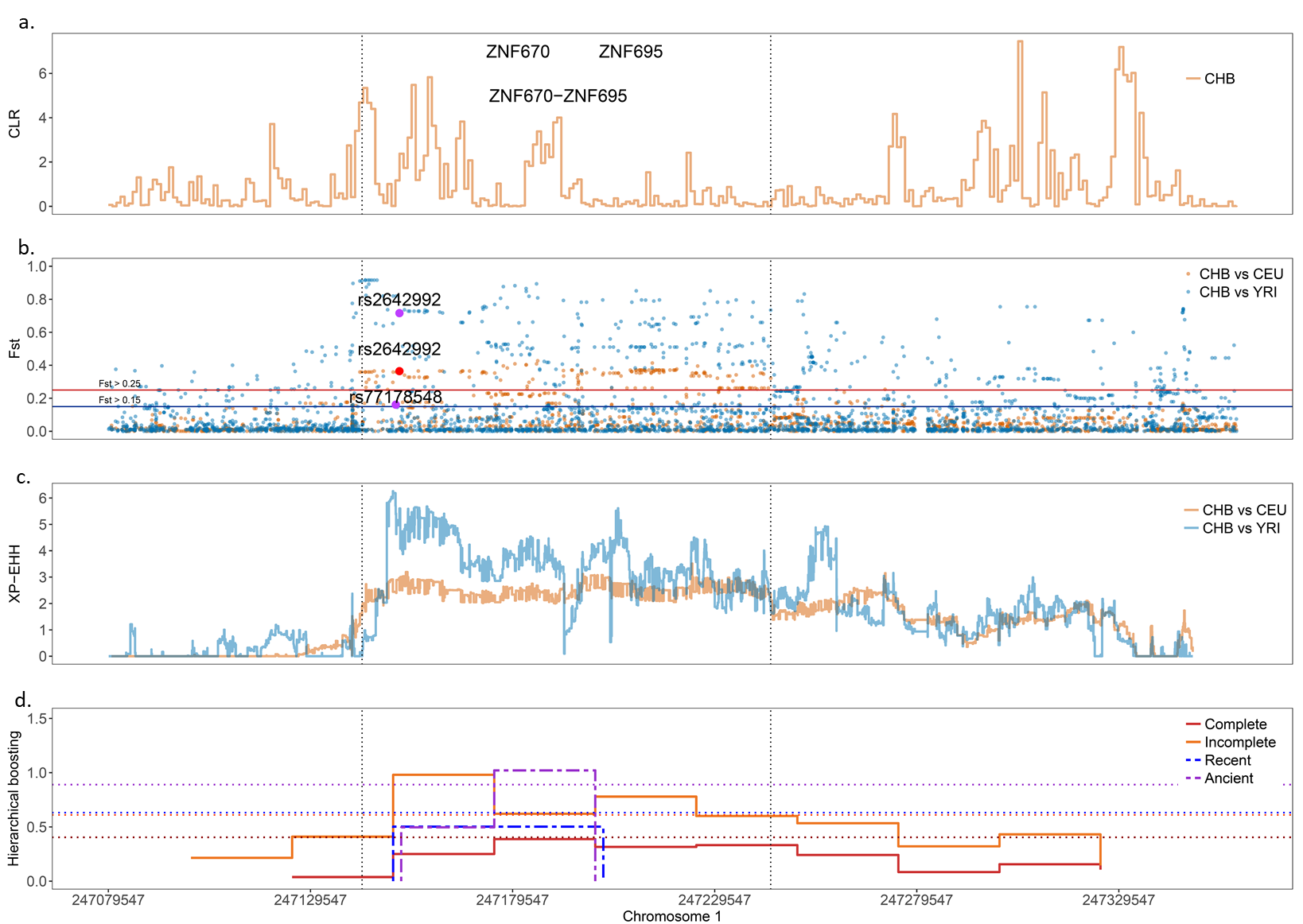


Figure S5. KRAB-ZNF gene cluster with EHH on the chromosome one of CHB. The candidate region spans around 100 kb (chr1:247142296-247243409; vertical pointed lines). This region contains two genes, *ZNF670* and *ZNF695*. Transcription of these two genes also produces a read-through transcript (*ZNF670*-*ZNF695*). Note that the scale on the y-axis differs between plots. **a.** High CLR scores indicating putative candidate regions for selection within this KRAB-ZNF cluster. **b.** F_st_ values show very high genetic differentiation between CHB and the other two populations (CEU and CHB) within the EHH region. The only two highly differentiated non-synonymous SNPs (rs77178548, rs2642992) located in regions coding for protein domains within this region are indicated with purple (CHB vs YRI) and red dots (CHB vs CEU). **c.** Very high scores in the XP-EHH track suggest the presence of long EHH when comparing CHB versus CEU and YRI. **d.** Results from the Hierarchical booting suggest that this region might have undergone an incomplete ancient selective sweep in CHB, about 29000 years ago (Pybus et al. 2015). Dotted horizontal lines indicate boosting thresholds: complete (red), incomplete (orange), incomplete ancient (violet), and recent (Blue).


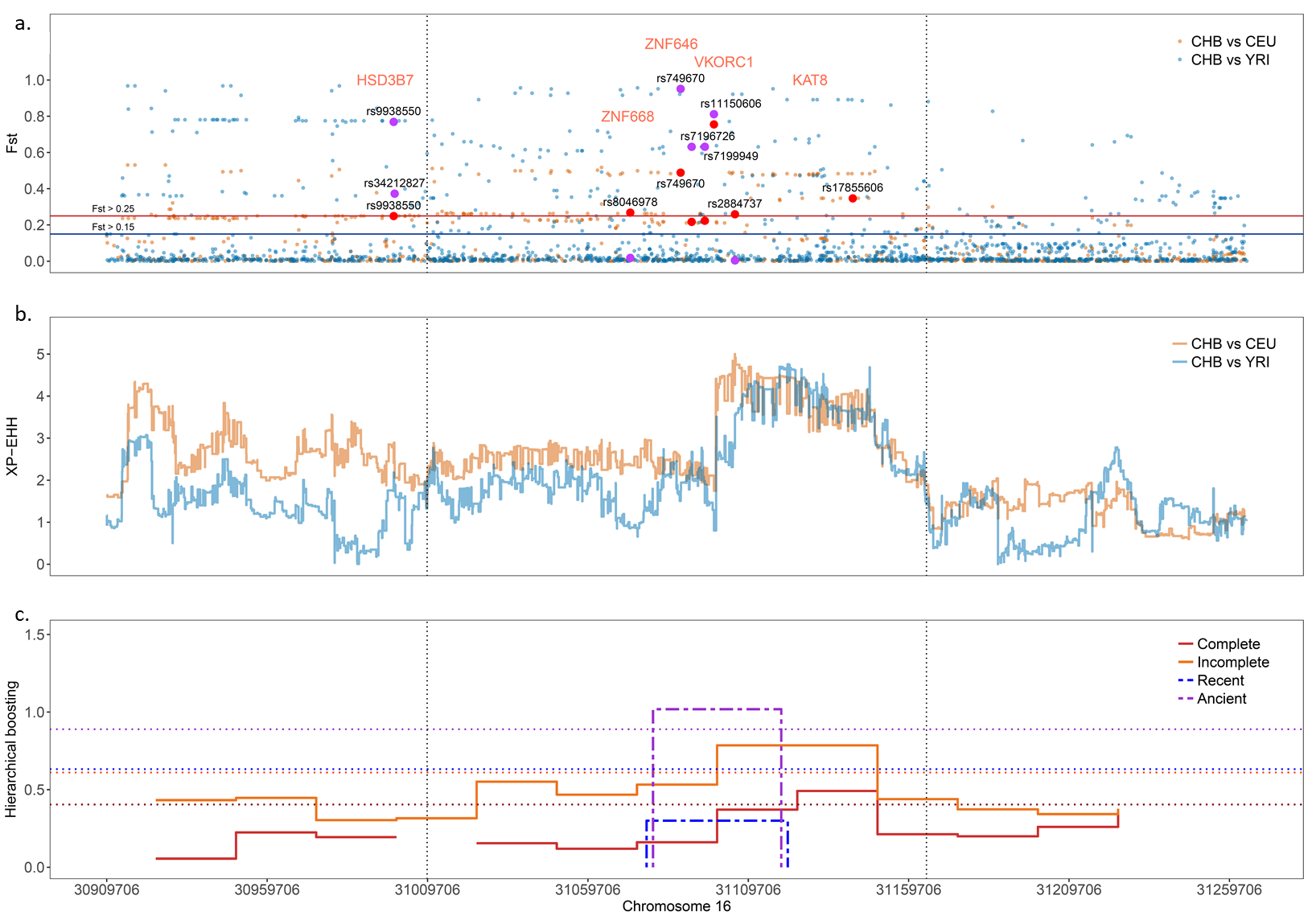


Figure S6. KRAB-ZNF gene cluster on chromosome 16 displaying very high genetic differentiation and EHH in CHB population. This extended haplotype spans 156 kbps (chr16:31009588-31165239). Note that the scale on the y-axis differs between plots. Genes with high to very high differentiated non-synonymous SNPs in regions coding for protein domains are represented by violet (CHB vs CEU) and red dots (CHB vs CEU). *ZNF646* carries the most differentiated SNP (Fst = 0.95), which suggests this variant as a very strong candidate target of positive selection. The haplotype decays in one SNP (rs74474326) at position (chr16:31009343) upstream of the EHH region (vertical pointed lines), and then continues for about 100 kb (XP-EHH track). This might be an artefact introduced by differences in the minor allele frequency. Therefore, we propose that this EHH very likely spans ~250 kb and not ~150 kb. Results from the Hierarchical booting suggests that this region might have undergone an incomplete ancient selective sweep in CHB, about 29000 years ago (Pybus et al. 2015). Dotted horizontal lines indicate boosting thresholds: complete (red), incomplete (orange), incomplete ancient (violet), and recent (Blue).

Despite it was initially suggested that the signature of a hard-selective sweep on the KRAB-ZNF gene cluster on chromosome 16 of CHB spans about 500 kb in all East Asian populations(Patillon et al. 2012), our results indicate that this region might be rather 250 kb smaller for CHB population. Six genes within this region (*ZNF646*, *KAT8*, *ZNF668*, *FBXL19, STX1B* and *VKORC1*) have been recently associated with obesity (Locke et al. 2015; Yazdi et al. 2015). One SNP located in the GRF gene *KAT8* is associated with body mass index in European individuals. Association and expression of quantitative trait loci suggest that this SNP also affects expression of the genes *ZNF646*, *VKORC1* and *ZNF668* (Locke et al. 2015; GTEx Consortium et al. 2017). In addition, four genes within this region have been associated with population-specific patterns in blood coagulation in European and Asian populations (*VKORC1*, *BCKDK*, *MYST1*, and *PRSS8*) (Patillon et al. 2012).

Figure S7. KRAB-ZNF gene cluster on chromosome six displaying very high genetic differentiation and EHH in CEU population. Colored dots correspond to the *F_ST_* empirical values obtained for SNPs when comparing CEU vs CHB (a, orange) and CEU vs YRI (b, blue). Grey dots correspond to *F_ST_* values obtained from simulated data (1000 simulations), as described in the Methods section. Although it is evident that high and very high genetically differentiated SNPs can be obtained from the simulated data when using a demographic model that fits to human historical events, none of the simulations we performed rendered total numbers of high and very high differentiated SNPs (*F_ST_* ) than those observed for the empirical data (p < 0.001).


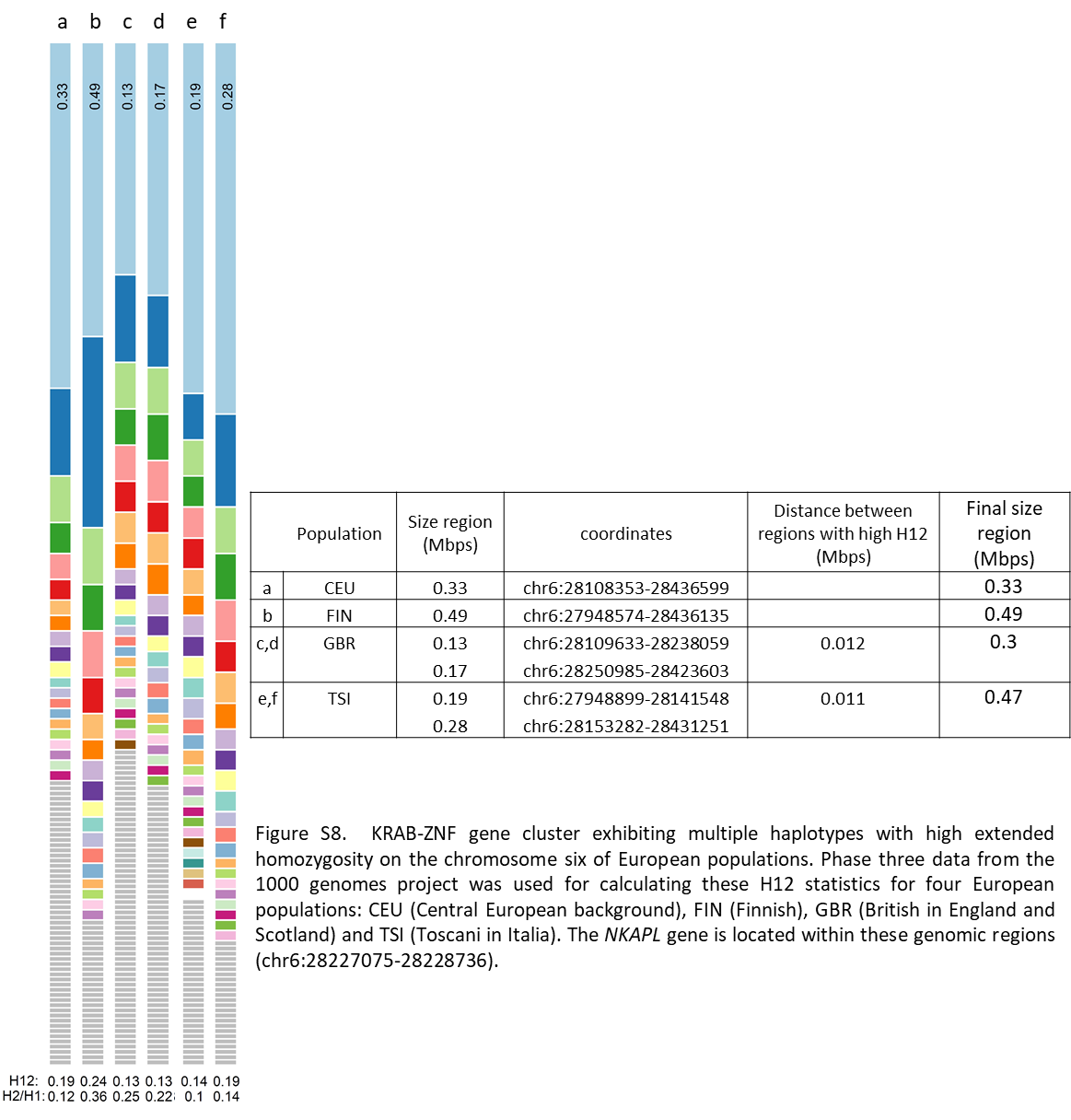


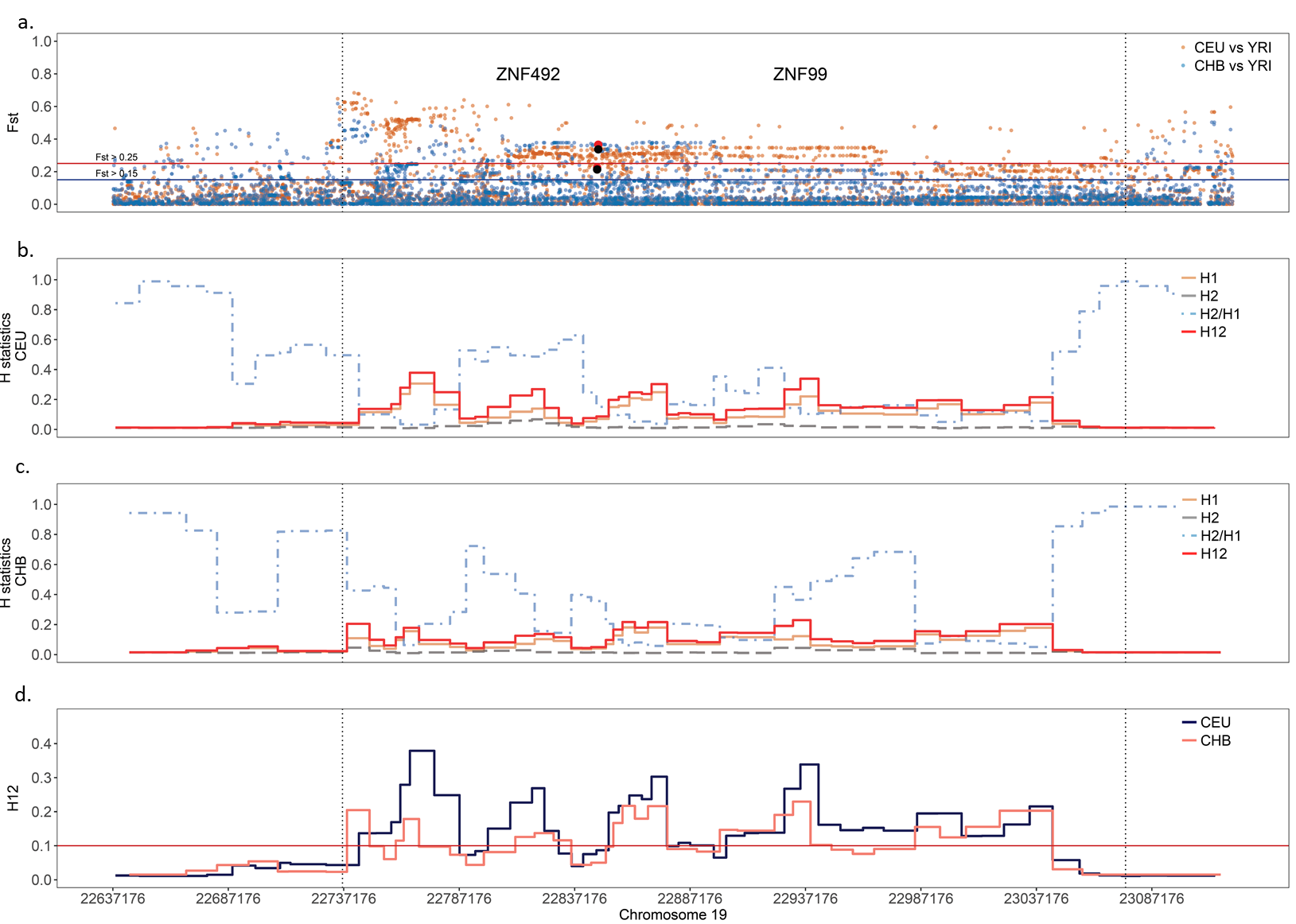


Figure S9. Human-specific positively selected region in two human populations, European (CEU) and Asian (CHB). It is located within a KRAB-ZNF gene cluster in chromosome 19 (chr19:22736627-23075779). This region (vertical pointed lines) spans 340 kb and contains the genes *ZNF492* and *ZNF99*. Note that the scale on the y-axis differs between plots. F_st_ values indicate moderated to high genetic differentiation between the Asian and the European populations when compared with Africans, respectively. H statistics shows a decrease of the H2/H1 ratio towards the middle of this gene cluster, which suggest the present of more than one haplotype at high frequency. H12 statistics increase in multiple regions beyond the H12 threshold (red line, H12 track, H12 > 0.1). This suggests that more than one haplotype may have undergone positive selection in CHB and CEU populations. Red (CHB vs YRI) and black (CEU vs YRI) dots indicate high genetically differentiated non-synonymous SNPs located in zinc fingers of *ZNF492* (rs138844698, rs141989264, rs144581197).


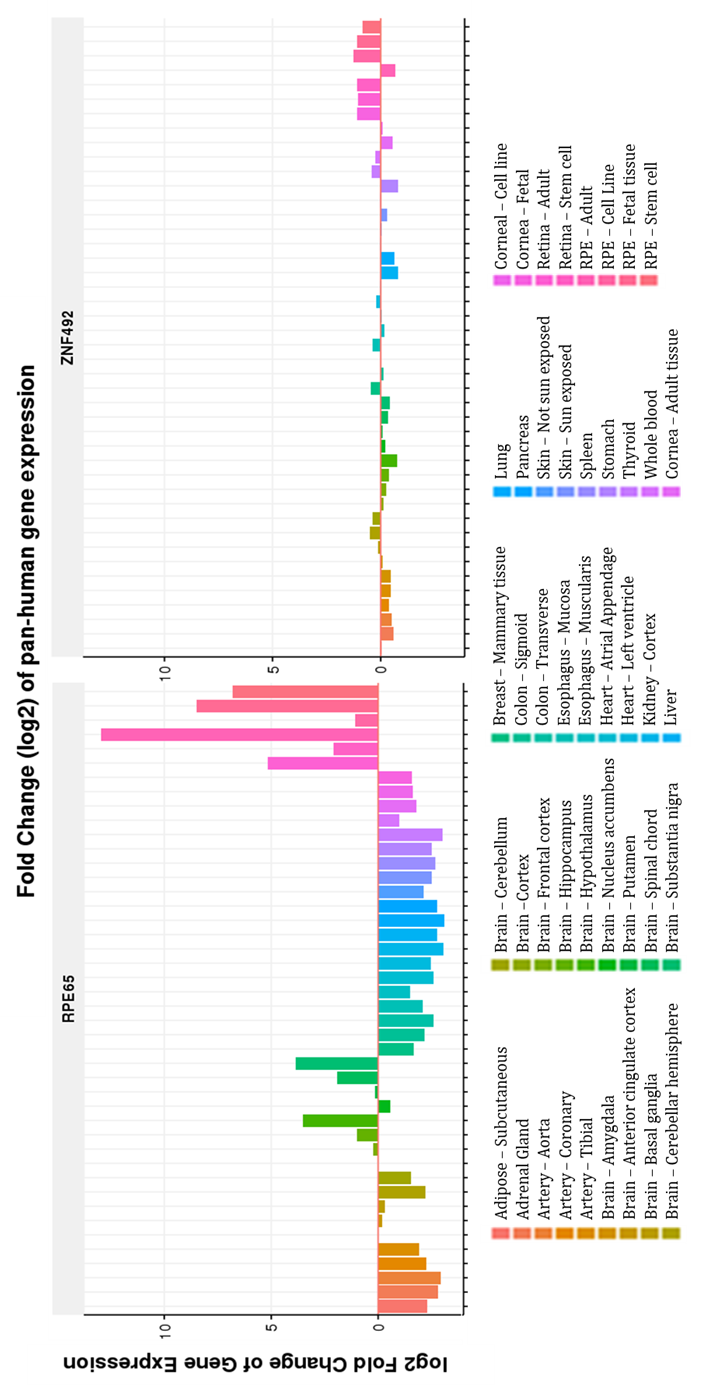


Figure S10. Histogram of the relative differential expression fold change (t-test) for *RPE65* and *ZNF492* across all tissues (*eyeIntegration database*) (Bryan et al. 2017). Pink to salmon colored bars show the variation in expression of these two genes across tissues from the human eye (cornea, retina and retinal pigment epithelium (RPE)). Although *ZNF492* displays lower average expression than *RPE65*, both genes display higher average expression in the human retina and eye tissues compared to most other tissues.


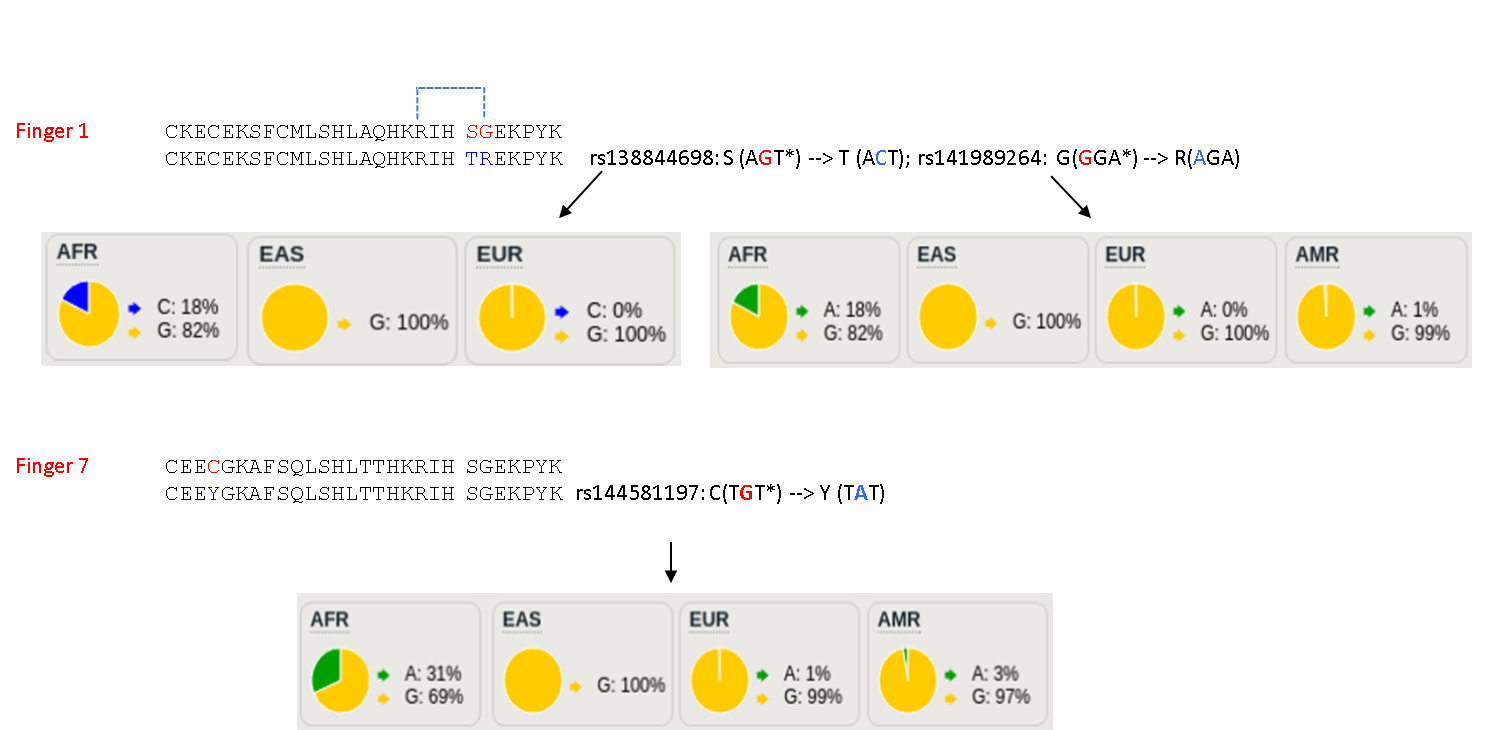


Figure S11. Non-synonymous SNPs that are introducing functional variation in two zinc fingers of the human-specific gene *ZNF492* at population-specific level. *ZNF492* is located in a candidate region for a soft selective sweep in CEU and CHB. (Top) Amino acid changes in the linker between the first and second zinc fingers of *ZNF492* might change the cap of the C terminus (C-cap) of the first finger (dashed blue line). Glycine is essential to complete the capping; thus, it can be expected that this change reduces the binding affinity of this finger to the DNA target sequence. (Bottom) Amino acid change in the highly conserved second cysteine residue disrupts the globular structure of the zinc finger and its stability due to the loss of zinc ion coordination with the two histidine residues (Laity et al. 2000). Pie charts represent the frequency in which each allele is found in AFR (Africans), EAS (East Asians); EUR (Europeans) and AMR (admixed American) populations (1000 Genomes Project Consortium 2012). (*) indicates the codon sequence found in CEU, CHB, Denisovan and Neandertal.

**References**

1000 Genomes Project Consortium. 2012. An integrated map of genetic variation from 1,092 human genomes. Nature. 491:56–65. doi: http://www.nature.com/nature/journal/v491/n7422/abs/nature11632.html#supplementary-information.

Garud NR, Messer PW, Buzbas EO, Petrov DA. 2015. Recent Selective Sweeps in North American Drosophila melanogaster Show Signatures of Soft Sweeps. PLOS Genet. 11:e1005004. doi: 10.1371/journal.pgen.1005004.

GTEx Consortium et al. 2017. Genetic effects on gene expression across human tissues. Nature. 550:204–213.

Laity JH, Dyson HJ, Wright PE. 2000. DNA-induced α-helix capping in conserved linker sequences is a determinant of binding affinity in Cys2-His2 zinc fingers1. J. Mol. Biol. 295:719–727. doi: 10.1006/jmbi.1999.3406.

Locke AE et al. 2015. Genetic studies of body mass index yield new insights for obesity biology. Nature. 518:197–206. doi: 10.1038/nature14177.

Patillon B et al. 2012. Positive Selection in the Chromosome 16 VKORC1 Genomic Region Has Contributed to the Variability of Anticoagulant Response in Humans. PLOS ONE. 7:e53049. doi: 10.1371/journal.pone.0053049.

Yazdi FT, Clee SM, Meyre D. 2015. Obesity genetics in mouse and human: back and forth, and back again. PeerJ. 3:e856. doi: 10.7717/peerj.856.
