# Supplementary material for: Genetic Variation in Human Gene Regulatory Factors Uncovers Regulatory Roles in Local Adaptation and Disease
Source: Genome Biol Evol. 2019 Jun 22;11(8):2178–93. doi: 10.1093/gbe/evz131 (PMC6685493; doi:10.1093/gbe/evz131)
Supplement: evz131_Supplementary_Data [file evz131_supplementary_data.zip › Supplementary_methods.docx]

**Supplementary methods**

Genetic variation in human gene regulatory factors uncovers regulatory roles in local adaptation and disease

Perdomo-Sabogal, A., and Nowick, K.

**Table of Contents**

Building the catalog of gene regulatory factors 2

Quantification of recombination rates differences 4

Testing for differences in GRFs length distribution ………………………………………………………………………4

Enrichment test for SNPs affecting protein domains 4

H1/H2 and H12 calculations 5

Demographic parameters used for simulating variation under neutral evolution 6

References 7

**Building the catalog of gene regulatory factors**

Using the extant inventories of genes involved in gene regulation (see table 1 in main document) we performed overlaps based on their stable identifiers (official gene symbols, Ensembl, UCSC and RefSeq IDs), batch coordinate conversions (liftOver) (Karolchik et al. 2014), manually inspection using different databases, and gene ontology for associating gene product attributes. We cross-validated the genes present in our catalog by using the information available in UCSC genome browser (Karolchik et al. 2014), UniProtKB (The UniProt Consortium 2015), and HUGO (Eyre et al. 2006) databases, among others. In cases where it was not possible to properly overlap particular regions or identifiers, mainly explained by changes in the names due to updates between genome versions, we lifted over the genomic coordinates between assemblies (Karolchik et al. 2014). We then manually explored the genomic regions for those cases in which all the previously mentioned strategies failed. In some cases, the use of non-official stable identifiers and failures when performing the liftOver limited our chances to track back all the information reported in the previous GRFs inventories. For all GRFs, including those listed in just one of the previous works, we additionally required that they were associated with a customized list of gene ontology (GO) terms associated with gene regulatory processes. We selected molecular and biological GO terms such as regulation of transcription, DNA-depending transcription, RNA polymerase II transcription cofactor and co-repressor activity, chromatin binding, remodeling, among 218 other terms (Supplemental_Table_S11). Then we sourced the GO information for the whole set of genes for the human reference GRCh37/hg19, in total 27,993 genes and around 80,900 transcripts, from the UCSC genome browser. This list also included information about genomic coordinates, several types of stable identifiers (i.e. Ensembl IDs, official gene symbols, UCSC IDs, RefSeq, among others).

For our own investigative purposes, the coordinates we report here have the smallest and the biggest coordinates of all transcripts reported for each entry, thus enabling us to define the whole genomic region for each GRF gene. In cases where the same gene had one to several non-overlapping transcripts, we decided to keep them separately. In such cases we added an additional tag at the end of the official gene symbol. This tag consists of a dash (-) followed by a capital letter from (A) to the number of non-overlapping transcripts. We also kept separately those GRF genes that have multiple copies located on the same chromosome, but do not overlap at all. In cases where alternative gene names were used to address conjoined genes, also known as fusion genes that produce read-through transcripts, we kept them separately. In addition and based on the results obtained by Huntley et al., (2006), we decided to keep several putative GRFs within our catalog, even when they are currently considered as pseudogenes, lincRNAs, or in the worst case scenario, they have a retired status. This is because Huntley and collaborators carried out manual curation (Huntley et al. 2006), which is why we consider their annotation as of high quality. Additionally, these genes show all the characteristics of protein coding genes, for instance, open reading frame without premature stop codons in the coding sequence (Nowick et al. 2011). For such cases, 22 KRAB-ZNF genes in total, the official gene symbols were tagged as follows: (+N) indicates the gene was reported as new in Huntley et al., (2006) but now is either considered a pseudogene, or has retired status in Ensembl and UCSC; (+NP) indicates that this gene is not considered a protein coding genes (but e.g. a ncRNA gene or pseudogene) in Ensembl and UCSC.

***Quantification of recombination rates differences***

We evaluated if the recombination rates of GRF genes significantly differ from those found for non-GRF genes by using the standardized recombination maps and rates from deCODE for the human genome reference GRCh37/hg19 (Kong et al. 2010). We quantified the distance between the empirical distributions for GRF and non-GRF genes by implementing a two-sample Kolmogorov–Smirnov test (KS).

***Testing for differences in GRFs length distribution***

Gene length can influence the number of SNPs per gene. We assessed if the distribution of the gene lengths between GRFs and non-GRFs was significantly different from each other to exclude the possibility of identifying length-dependent artifacts as candidates for positive selection. We performed a Spearman rank correlation test to measure if there was statistical dependence between gene length and the rank scores obtained for each of the four tests.

**Enrichment test for SNPs affecting protein domains**

Using information from the SNP based tests (XP-EHH and FST), we explored if higher scores were mostly occurring on SNPs located in functional domains, and if these correspond to synonymous or non-synonymous SNPs. We first identified the SNPs and their types (synonymous/non-synonymous) in all genes from all GRF classes. We then evaluated if non-synonymous SNPs were more commonly found in interacting or non-interacting domains (Fisher Exact test).

***H1/H2 and H12 calculations***

To detect if the KRAB-ZNF gene clusters that we detected as candidates for selection carry one or more haplotypes with high frequency EHH, we implemented H12 methods, which are based on the haplotype homozygosity (Garud et al. 2015). We obtained the genetic information (1000 genomes project) for those chromosomes where the 32 KRAB-ZNF gene clusters we detected as putative selected regions are located (Huntley et al. 2006) for CEU, CHB and YRI populations. We filtered variants that had a quality of the assigned genotype (GQ) smaller than 20. Since the calculation of the H12 statistics requires the definition of a window size in terms of SNPs, that such window is sensitive to linkage disequilibrium (LD), and that LD varies between human populations, we calculated the LD decay with physical distance by means of r^2^ (Hill & Robertson 1968) for each population. In all three populations we measured where the LD decays below r^2^ = 0.1 (between 20 and 50 kb). Subsequently, we calculated the mean number of SNPs for windows of 50 kb genome wide for each population and used it as input argument for H1/H2 an H12. We used 50 SNPs as the distance between the center of the analyses, and H12 > 0.1 as the threshold for identifying peaks of H12 chromosome–wide. We then used the Cosi2 simulator (Shlyakhter et al. 2014), as described in the Methods section, to compare the empirical results versus the simulated data.

**Demographic parameters used for simulating variation under neutral evolution**

This is an example of the parameters file generated for simulating data under evolutionary scenarios that are consistent with human demographic scenarios for three populations (CEU, CHB, and YRI) as described by Schaffner et al. (2005) and using Cosi2 simulator (Shlyakhter et al. 2014).

## recosim, a tool included in Cosi2, was previously run to generate the recombination rates that will be used as input files (model.test file). recosim uses the recombination rates from the deCODE genetic maps produced by Kong et al. (2002, 2010).

# sequence features

length 1000000

mutation_rate 1.5e-8

recomb_file model.test

gene_conversion_relative_rate 4.5e-9

# population info

pop_define 1 european

pop_define 2 asian

pop_define 3 african

# european

pop_size 1 100000

sample_size 1 170

# asian

pop_size 2 100000

sample_size 2 136

# african

pop_size 3 100000

sample_size 3 156

#Demographic events

pop_event change_size "agriculture - african" 3 200 24000

pop_event change_size "agriculture - european" 1 350 7700

pop_event change_size "agriculture - asian" 2 400 7700

pop_event bottleneck "african bottleneck" 3 1997 .008

pop_event bottleneck "asian bottleneck" 2 1998 .067

pop_event bottleneck "european bottleneck" 1 1999 .02

pop_event split "asian and european split" 1 2 2000

pop_event bottleneck "OoA bottleneck" 1 3499 .085

pop_event split "out of Africa" 3 1 3500

pop_event change_size "african pop size" 3 17000 12500

### To simulate scenarios under non-neutral evolution, the next line can be adjusted. Parameters such as final frequency and the start and end time can be customized as suggested by Pybus et al. (2015).

#pop_event sweep "hard sweep " 1 201 0.033 0.5 0.3

**References**

Garud NR, Messer PW, Buzbas EO, Petrov DA. 2015. Recent Selective Sweeps in North American Drosophila melanogaster Show Signatures of Soft Sweeps. PLOS Genet. 11:e1005004. doi: 10.1371/journal.pgen.1005004.

Hill WG, Robertson A. 1968. Linkage disequilibrium in finite populations. Theor. Appl. Genet. 38:226–231. doi: 10.1007/BF01245622.

Huntley S et al. 2006. A comprehensive catalog of human KRAB-associated zinc finger genes: insights into the evolutionary history of a large family of transcriptional repressors. Genome Res. 16:669–677. doi: 10.1101/gr.4842106.

Kong A et al. 2002. A high-resolution recombination map of the human genome. Nat. Genet. 31:241–247. doi: 10.1038/ng917.

Kong A et al. 2010. Fine-scale recombination rate differences between sexes, populations and individuals. Nature. 467:1099.

Pybus M et al. 2015. Hierarchical boosting: a machine-learning framework to detect and classify hard selective sweeps in human populations. Bioinforma. Oxf. Engl. 31:3946. doi: 10.1093/bioinformatics/btv493.

Schaffner SF et al. 2005. Calibrating a coalescent simulation of human genome sequence variation. Genome Res. 15:1576–1583. doi: 10.1101/gr.3709305.

Shlyakhter I, Sabeti PC, Schaffner SF. 2014. Cosi2: an efficient simulator of exact and approximate coalescent with selection. Bioinformatics. 30:3427–3429. doi: 10.1093/bioinformatics/btu562.
